# Fe Incorporation in Ni-Based Layered Hydroxides: Implications for Oxygen Evolution Electrocatalysis

**DOI:** 10.1021/acs.inorgchem.5c02786

**Published:** 2025-11-21

**Authors:** Camilo Jaramillo-Hernández, Alvaro Seijas-Da Silva, Vicente B. Vert, Martin Mizrahi, Antonio Leyva-Pérez, Gonzalo Abellán

**Affiliations:** † Instituto de Ciencia Molecular (ICMol), Universidad de Valencia, Catedrático José Beltrán 2, 46980 Paterna, Valencia Spain; ‡ Instituto de Investigaciones Fisicoquímicas Teóricas y Aplicadas (INIFTA) Departamento de Química, Facultad de Ciencias Exactas Universidad Nacional de La Plata, CCT La Plata- CONICET Diagonal 113 y 64, 1900 La Plata, Argentina; § Facultad de Ingeniería, 28228 Universidad Nacional de La Plata, calle 1 esq. 47, 1900 La Plata, Argentina; ∥ Instituto de Tecnología Química, Universidad Politècnica de València−Agencia Estatal Consejo Superior de Investigaciones Científicas, Avda. de los Naranjos s/n, 46022 Valencia, Spain

## Abstract

Alkaline water electrolysis (AWE) is a promising hydrogen production method but faces challenges with the sluggish oxygen evolution reaction (OER), which requires high voltages. Nickel-based layered hydroxides (LHs) are effective earth-abundant OER catalysts, though Fe incorporation from electrolyte impurities significantly enhances their performance. This study systematically examines Fe impurity incorporation in Ni-based LH phases: α-Ni-LH, β-Ni-LH, and NiAl- and NiFe-layered double hydroxides (LDHs). Two incorporation methods were explored: a standard electrolyte purification process and an electrochemical activation approach. Electrochemical activation is more effective, and expanded phases have more affinity to allocate Fe. Incorporation experiments suggest a partial transformation of NiAl into NiFe-like LDH, which exhibits a superior electrocatalytic performance. Spectroscopical techniques suggest that the Fe incorporated in the NiAl LDH could be structural due to synergy with the concomitant leaching of Al in the electrolyte. For pristine NiFe-LDH, these treatment strategies proved ineffective, suggesting that such approaches are unsuitable for optimized compositions. Furthermore, the process is highly dependent on the Fe impurity concentration in the electrolyte. This work highlights the role of the initial LH phase in determining structural Fe incorporation, providing insights for designing efficient electrodes in AWE. It also emphasizes the need for strict control of the electrolyte to optimize catalyst performance.

## Graphical abstract

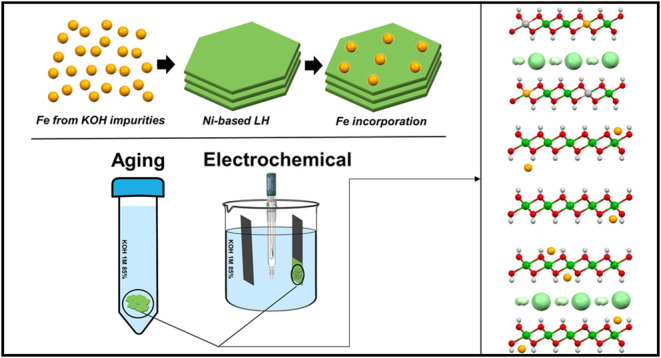

## Introduction

Alkaline water electrolysis is regarded as one of the most sustainable methods for producing hydrogen.^1–3^ However, the oxygen evolution reaction (OER), which stands as the bottleneck for this technology due to its slow kinetics, thus demanding high voltages. This process leads to a significant loss in energy efficiency within water-splitting systems, requiring the implementation of better catalysts to lower the energy barriers.^4–6^ Over the past decade, considerable efforts have been dedicated to developing outperforming electrocatalysts derived from abundant resources, as opposed to traditional benchmarks based on platinum group metals (PGM), reducing costs and minimizing raw material supply problems. Among the different non-precious-metal OER catalysts reported in the literature, nickel-based materials have risen as one of the most widely adopted.^7–9^

Layered hydroxides (LHs) have proven to be exceptional electrocatalysts for the OER standing out about all the NiFe-based layered hydroxides.^9–11^ Moreover, their chemical versatility has facilitated the exploration of modified compounds to enhance OER activity by tuning different phases,^12–14^ morphologies,^15^ interlayer anions,^16–19^ or cationic compositions.^8,20^ The observed catalytic improvement is the result of increased surface areas, enhanced OH– adsorption ability, and the intrinsic reactivity of the electroactive sites. Besides their impressive electrochemical performance, these materials are also cost-effective, and composed of nongeolocalized abundant elements, which make them practical for water electrolysis applications.^21–25^ Currently, the most promising phases for OER electrocatalyst cannot be obtained through conventional synthetic methods, and their scale-up remains elusive, with few exceptions.^26–29^

When evaluating the electrochemical properties of the catalyst in alkaline water electrolysis, the presence of iron (Fe) impurities—originating from the electrolyte (NaOH and KOH) or leached from system components such as pipes, glassware, and reactors—has become a high-importance topic due to the extrinsic catalytic performance enhancement observed.^30–34^

This boosting, also observed in different families of catalyst for OER such as perovskites,^35,36^ gold-based electrodes,^37–39^ metal (hydr)­oxides,^30,40^ and Ni-based metal X-ides,^41–43^ among others, can reach up to a 200-fold increase in the electrochemical performance. The enhancement is highly dependent on the catalyst used, its affinity for Fe, and the electrochemical process involved. Indeed, the in situ incorporation of Fe in typical Ni-based materials used as electrocatalysts could lead to the formation of new and more electroactive phases that could hinder real performance in novel chemistries. As a result, efforts have emerged to purify these electrolytes used for characterization employing materials like Ni collectors and Ni hydroxides,^44^ besides the use of electrochemical techniques for being applied in high-quality electrochemical tests.^45,46^ The review by Li et al. summarizes the effects of Fe incorporation in various materials, through different approaches, in a systematic way, demonstrating that the enhancement of the electrochemical properties can be intended as well.^34^

Inspired by these reports and the techniques proposed, in this work, we employ two previously reported methods for electrolyte purification to explore the extent of Fe incorporation in three different Ni-based layered materials. Besides a mechanical method,^44^ where Ni-based layered materials are aged against nonpurified KOH, an in situ electrochemical approach was also conducted, according to different works that claim that the activation processes for some materials using a nonpurified KOH leads to a Fe incorporation.^45,46^

The results demonstrate that the electrochemical approach is the most effective technique for iron incorporation into Ni-based hydroxides. Among the different phases studied for the Ni-based layered phases, the expanded phases exhibit the highest affinity for incorporating Fe at lower potentials. Subsequent electrochemical characterization suggests that the NiAl phase may incorporate Fe structurally within the layer due to the dissolution of Al in the basic medium, resulting in an improved electrochemical performance. Moreover, pristine NiFe-LDH did not benefit from the studied incorporation processes, suggesting that such approaches are unsuitable for LH electrocatalysts with an optimized Fe content. These incorporation methods are highly dependent on the quantity of impurities present in the electrolyte, leading to variable results when relying solely on impurities in commercial electrolytes. Overall, this work provides a systematic study of the impact of Fe incorporation in Ni-based layered materials on their electrochemical performance in alkaline water electrolysis. It paves the way for the straightforward preparation of NiFe-based efficient electrocatalysts and emphasizes the importance of controlling the concentration of relevant transition metal cations in electrolytes.

## Experimental Section

### Chemicals

Aluminum chloride hexahydrate (AlCl_3_·6H_2_O), (NiCl_2_·6H_2_O), urea, hexamethylenetetramine (HMT), glycidol (Gly), acetylene black, and Nafion (117 solution) were purchased from Sigma-Aldrich.

Potassium hydroxide (KOH) at 85% was purchased from Supelco, Thermo Fisher, and Sigma-Aldrich, and at 99.98% from Thermo Fisher. Ethanol absolute (EtOH) was purchased from Panreac. All chemicals were used as received. Milli-Q water was obtained from Millipore Milli-Q equipment. NiFe-LDH was purchased from Matteco Team S.L.

### LH Synthesis

Samples were obtained through specific synthetic protocols for each phase, as described below. In all the cases, solids were separated for the mother liquors by filtration, washed three times with water, water:ethanol mixture, and finally with ethanol. Samples were dried at room temperature and kept in desiccators for further characterization.

### Synthesis of α-LH

α-Ni LH was synthesized by employing the Epoxide Route^47,48^ following the protocol reported by Arencibia et al.^49,50^ Typically, precipitation is driven by the reaction taking place between chloride and Gly at room temperature for 48 h, in an aqueous solution containing initial concentrations fixed to [NiCl_2_·6H_2_O] = 10 mM, [NaCl] = 80 mM, [Gly] = 400 mM.

### Synthesis of β-LH

β-Ni LH sample was synthesized by employing HMT as an alkalinization reagent following the protocol reported by Liang et al.^51^ Typically, β-LH precipitation is driven by the hydrolysis of HMT at ca. 97 °C for 5h, under an inert atmosphere, in an aqueous solution containing initial concentrations fixed to [NiCl_2_·6H_2_O] = 7.5 mM and [MHT] = 45 mM.

### Synthesis of NiAl LDH

NiAl LDH was synthesized following a hydrothermal route by employing urea as an alkalinization reagent following the protocol reported by Liu et al.^52^ Typically, LDH precipitation is driven by the hydrolysis of urea at ca. 97 °C for 48 h, under inert an atmosphere, in an aqueous solution containing initial concentrations fixed to [NiCl_2_·6H_2_O] = 10 mM, [AlCl_3_·6H_2_O] = 5 mM, [urea] = 70 mM. I

### Fe Incorporation Approaches

#### Aging Incorporation

Based on the work of Marquez et al.,^44^ in a 50 mL Falcon tube, 20 mg of a Ni-based layered hydroxide was introduced. The hydroxide was subjected to a thorough washing process with 4 mL of 1 M KOH solution (purity of 85%). The mixture was vigorously agitated by using a vortex mixer. Subsequently, an additional 20 mL of the same 1 M KOH solution was added to the Falcon tube, followed by a 10 min sonication period. The Falcon tube containing the hydroxide suspension was once again vigorously shaken in a vortex, subjected to centrifugation at 8000 rpm for 10 min, and then shaken again. The resulting suspension was left aging for a duration of 48 h. After that time, the solids were recuperated by filtration and dried in a desiccator.

#### Electrochemical Incorporation

The electrodes, which were prepared as delineated in the electrochemical characterization section, were carefully positioned within a three-electrode cell configuration. This setup featured a Ag/AgCl reference electrode, complemented by a slightly larger Carbon Paper counter electrode. Subsequently, a total of 2000 cyclic voltammetries were conducted within a voltage range spanning from −0.2 to 0.55 V vs reference, employing a 1 or 6 M KOH electrolyte (with an 85% purity level). Following this initial set of experiments, the electrolyte was substituted with a meticulously purified 99.9% 1 M KOH solution.

The first electrochemical incorporation experiments were performed using a KOH 85% provided by Supelco, while the following experiments were performed using a KOH 85% ultrapure, from Thermo Fisher.

#### KOH Purification

To purify the electrolyte (KOH 1 M 99.98% purity), Ni fibers (BEKIPOR 2Ni18–0.25, Bekaert, 99.9% purity) were used as both working and counter electrodes for a prolonged electrolysis process lasting 1 day at high current densities. This approach was motivated by previous reports.^45,46^

### Electrochemical Characterization

#### Electrode Preparation

Inks for the Ni-based layered hydroxides were prepared using 3 mg/mL solid and 5 μL/mL Nafion 5% in ethanol. The dispersion was stirred overnight to obtain a well-dispersed suspension. In the case where it was necessary, the dispersion was also sonicated. The ink was deposited on Carbon Paper collectors with an area from 3 to 5 cm^2^ by spray coating (using an airbrush from Harder Evolution), obtaining an average mass loading of 0.60 mg/cm^2^.

#### Electrochemical Measurements

Electrochemical tests were performed in a three-electrode cell equipped with a carbon paper electrode acting as the working electrode with an electrode size of 1 cm^2^ and a platinum electrode as counter electrode of 2 cm^2^. As the reference electrode, a silver–silver chloride (Ag/AgCl (3 M KCl)) was used.

All potentials were converted by referring to the oxygen evolution reaction overpotential. Gamry 1000E potentiostat/galvanostat was controlled by Gamry. Linear sweep voltammetry (LSV) measurements were carried out at 5 mV/s in a 1 M KOH aqueous solution. Prior to this, 30 cyclic voltammetry measurements were performed at 50 mV/s to activate the material.

#### Chemical and Structural Characterization

Induced coupled plasma mass spectroscopy (ICP-MS) was carried out by using an Agilent Technologies. ICPMS7900. Samples after the electrochemical characterization, in carbon paper, were pretreated in a digestion microwave. All samples of KOH were measured using a solution of 1 M.

Powder X-ray powder diffraction (PXRD) patterns were obtained employing a PANalytical Empyrean X-ray platform with a capillary platform or a Bruker D8 ADVANCE A25 with surface platform and copper radiation (Cu Kα = 1.54178 Å). Measurements were carried out in triplicate in the 2-θ range 2–70° by employing a step size of 0.02 °/step with an integration time of 1 s.

Scanning electron microscopy with energy-dispersive X-ray spectroscopy (SEM-EDX) data was acquired using a SCSIOS 2 FIB-SEM, with a beam energy of 10 keV. The samples on carbon paper were directly investigated without any surface coating.

Transmission electron microscopy images were acquired using a Hitachi HT 7800 TEM, with a beam energy of 100 keV. The samples were deposited on Cu grids prior to the measurement.

Individual point Raman spectra were carried out using a Horiba LabRAM HR evolution, employing a red laser (633 nm) in the 100–1000 cm^–1^ range. For the acquisition of all Raman spectra, a 50× Objective with a 600 mm^–1^ grating was employed. Measurements were performed at least five times at 1.25 mM laser power, with an acquisition time of 20 s.

X-ray photoelectron spectroscopy (XPS) measurements were recorded on a Thermo Scientific K-Alpha X-ray Photoelectron Spectrometer. Al Kα X-ray radiation was employed as an X-ray source (1486.6 eV). For all the elements, more than 100 spectra were recorded employing a step of 0.1 eV with a focused spot higher than 400 μm. XPS data were analyzed with Thermo Avantage v5.9912 software. Additional XPS measurements were performed using a SPECS spectrometer equipped with a Phoibos 150 MCD-9 analyzer using nonmonochromatic Mg Kα (1253.6 eV) X-ray source working at 50 W. As reference for the peak positions in the XPS spectra, the C 1s peak has been set at 284.5 eV.

#### X-ray Absorption Spectroscopy

XANES and EXAFS spectra at the Ni and Fe K-edges were recorded at the BL16-NOTOS beamline of the ALBA Synchrotron. The measurements were performed at room temperature in the fluorescence mode. The samples were deposited onto carbon paper and measured before and after the treatments.

A Si(111) double-crystal monochromator was used to obtain a monochromatic incident beam over the sample. The intensity of the incident X-rays was measured using an ionization chamber, and the fluorescence signal was detected using a 13-element SDD detector. XAS spectra were collected in fly-scan mode for both edges: from 7000 to 7500 eV for the Fe K-edge and from 8200 to 9300 eV for the Ni K-edge, with energy steps of 0.3 eV in both cases. The incident photon energy was calibrated using the first inflection point of the Fe K-edge (7112 eV) and Ni K-edge (8333 eV) from reference foils of metallic Fe and Ni, respectively. For each sample, three spectra were taken with exposure times of 4 min each one to later be averaged. XANES data treatment was performed by subtracting the pre-edge background, followed by normalization by extrapolation of a quadratic polynomial fitted at the post-edge region of the spectrum using ATHENA AUTOBK background removal algorithm.^53^

The quantitative analysis of the EXAFS results was performed by modeling and fitting the isolated EXAFS oscillations. The EXAFS oscillations χ­(k) were extracted from the experimental data with standard procedures by using the Athena program. The k^2^ weighted χ­(k) data, to enhance the oscillations at higher k, were Fourier transformed. The Fourier transformation was calculated using the Sine filtering function. EXAFS modeling was carried out using the LARCH software.^54^

Theoretical scattering path amplitudes and phase shifts for all paths used in the fits were calculated using the FEFF9 code.^55^ The k-range was set from 2.5 to 12.0 Å^–1^. The passive reduction factor S_0_^2^ was restrained to 0.82 for Ni fitting. This value was obtained from the fitting of a standard metallic Ni foil with the coordination numbers constrained to those corresponding to its structure.

## Results

Two different approaches were employed to incorporate Fe into Ni-based layered hydroxides (Scheme 1). The first approach (Scheme 1A) is an adaptation of the purification method proposed by Marquez et al.,^44^ where a nonpurified KOH (1 M) is aged in contact with a nickel salt to form a Ni-hydroxide. This Ni-hydroxide further incorporates Fe impurities between its layers. In this adaptation, intentionally synthesized Ni-based layered hydroxides were used instead of nickel salts to study the incorporation of iron in these layered materials. This approach is referred to as “aging incorporation” throughout the text.

**Scheme 1:**
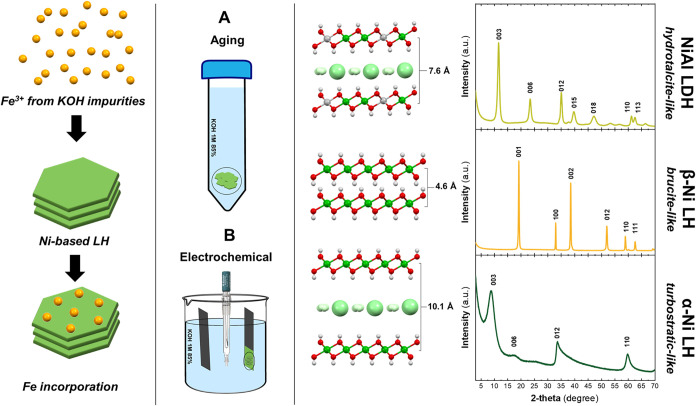
Proposed Methodologies for the Fe Incorporation in Ni-Based Layered Hydroxides^a^

a Aging and electrochemical incorporations were performed under KOH 85% 1 M.

The second approach (Scheme 1B) involves electrochemical incorporation based on material activation through cyclic voltammetry applied in ranges below the OER over 2000 cycles. This will be denoted as “electrochemical incorporation” henceforth. The as-synthesized samples and those obtained after applying both Fe incorporation approaches were subjected to electrochemical measurements, including cyclic voltammetries (CVs) and linear sweep voltammetries (LSVs).

The aim of this work is to explore how these methods of Fe incorporation interact with Ni-based layered hydroxides without necessarily focusing on improved OER catalyst performance. For this purpose, electrochemical incorporation and subsequent electrochemical characterizations were performed by using carbon paper as the supporting electrode. This type of electrode allows the performance of post-mortem characterization of the materials. Unlike typical Ni-based electrodes used in OER, the absence of metal in carbon paper eliminates interfering signals, allowing for more accurate observations and interpretations, even though it may exhibit slightly lower electrochemical performance.^20^

To perform these incorporation methods, a prior study of the impurities present in five different commercial KOH samples was conducted using inductively coupled plasma mass spectrometry (ICP-MS) to select the one most suitable for our purpose. The samples were prepared as 1 M solutions for comparability. The KOH sample labeled KOH 1 (with 85% purity) had the highest amount of Fe impurities and was selected for the incorporation approaches (see Figure S1).

Fe incorporation tests were conducted on three different Ni-based layered hydroxides (LHs), including the Brucite-like (β-Ni LH), Al-containing hydrotalcite-like (NiAl LDH), and turbostratic-like (α-Ni LH) phases (see Scheme 1, right side) to validate their ability to allocate Fe ions. The choice of aluminum in the hydrotalcite-like phase is due to its nonactive nature, ensuring that the observed effects are primarily attributed to the divalent metal, which allows for a more straightforward comparison between the three phases. Moreover, in previous studies, it was observed that under similar conditions to those proposed for the electrochemical incorporation experiments (1 M KOH at room temperature), the NiAl 2:1 sample remained stable during long-term measurements (24 h).^13^

In the nonexpanded β-LH phase, metallic cations are exclusively situated in octahedral environments M^II^(O_*h*_), resulting in basal space distances (*d*_BS_) below 5 Å.^56,57^ One of the most relevant materials of the LH family are the layered double hydroxides (LDHs), also known as anionic clays.^58^ LDHs consist of positively charged layers containing divalent M^II^(O_*h*_) and trivalent M^III^(O_*h*_) cations. Due to the excess of charge, anions and solvent molecules are incorporated into the interlayer space, expanding the dBS to values greater than 7 Å. The electrostatic interaction between the anions and the layers allows for the synthesis of various LDHs through anion exchange reactions. This versatility results in numerous intriguing materials with diverse applications, with a primary focus on energy systems.^59–61^

Finally, the α-LH phases are expanded structures with anions located in the interlayer space.^62,63^ The specific crystallographic structure and anion-sheet interaction in α-LH phases are highly dependent on the nature of the divalent cations, which can adopt octahedral­(O_*h*_) or tetrahedral­(T_*d*_) environments.^12^ For example, Co-based LHs exhibit this variability.^64–66^ However, Ni-based α-LHs feature a turbostratic layered hexagonal structure, exclusively containing Ni^II^(O_*h*_).^56,67^ Notably, even in the absence of trivalent cations, this layered compound exhibits anion exchange properties like LDHs due to the presence of nickel vacancies along the octahedral environment.^68^

In Figure S2, powder X-ray diffraction (PXRD) patterns are presented, highlighting the differences among the three studied phases. These phases are consistent with the corresponding ICDD reference data reported in the literature (ICDD 38–0715 for α-Ni LH, ICDD 14–0117 for β-Ni LH, and ICDD 15–0087 for NiAl LDH).^69–75^ However, in the case of α-Ni LH, a noticeable shift of the low-angle reflections is observed compared to the ICDD references, attributed to the specific room-temperature synthetic conditions that promote a less crystalline character. This is evidenced by the broad bands corresponding to the main reflections at low 2θ values, but remain in agreement with the α-Ni LH reported previously for this synthetic approach.^13,49^

Prior to any iron incorporation, an electrochemical analysis of the as-synthesized phases was performed, employing a purified 1 M KOH solution as electrolyte (see the Experimental Section for further details). An ICP-MS analysis evaluating the amount of Fe and Ni before and after the purification method for the electrolyte is presented in Figure S3, exhibiting a decrease of Fe impurities in the electrolyte. In Figure 1, a summary of the electrochemical characterizations of as-synthesized Ni-LHs is presented, including the activation process of each material by CVs and a comparison in the OER performance by LSVs. Figure 1A illustrates the activation process for the different materials, with the initial cycle depicted in black and the final cycle represented in the corresponding color (lighter colors showed the electrochemical evolution during the activation process).

**Figure 1:**
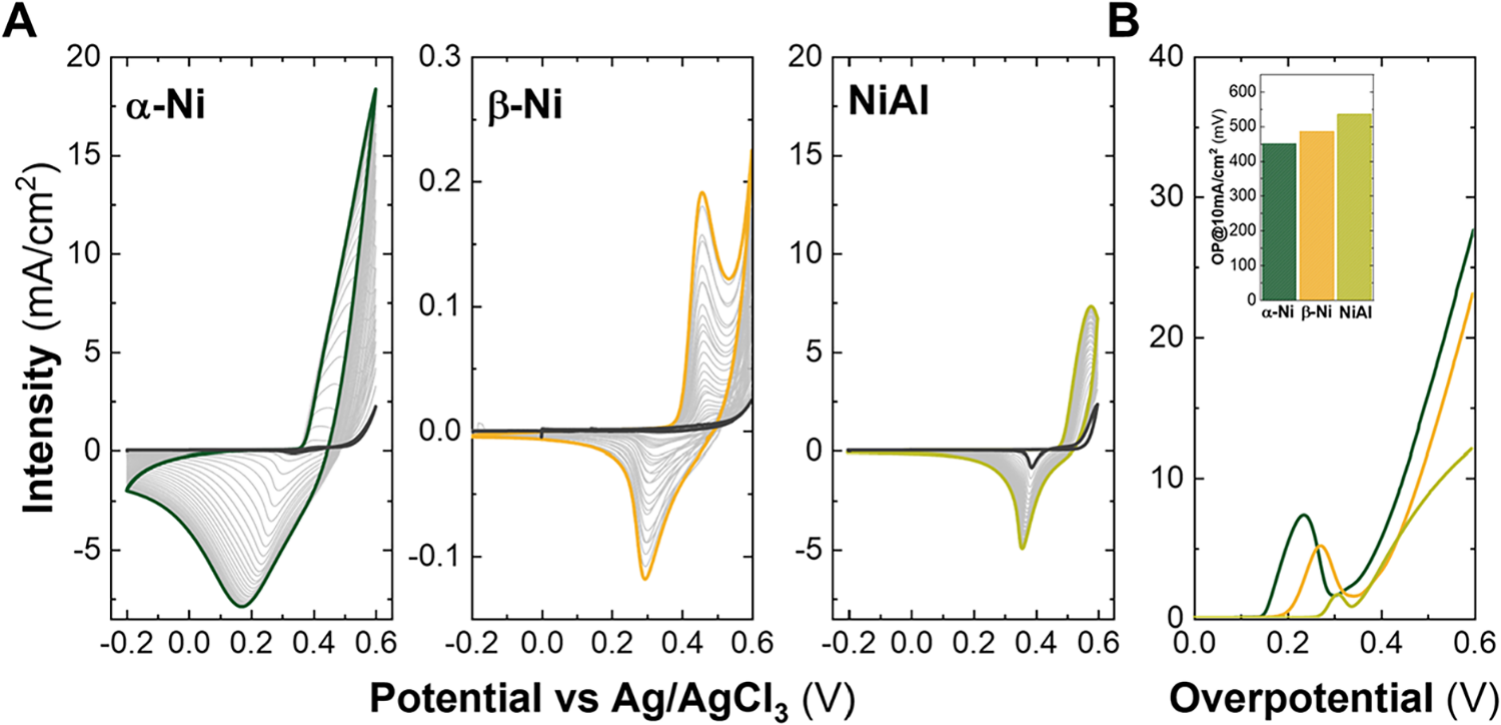
Electrochemical characterization in purified 1 M KOH of the as-synthesized samples prior to iron incorporation treatments: Cyclic voltammetries for the samples α-Ni LH, β-Ni LH, and NiAl LDH in (A). Linear sweep voltammetry curves measured at 5 mV/s (B) and overpotential values required for a current density (values at 10 mA/cm^2^ depicted in the inset in (B)).

In all cases, a typical activation process of layered hydroxides is observed: the first cycle exhibits low current density values, while subsequent cycles display a gradual increase. This behavior is attributed to the poor electrical conductivity of as-synthesized LHs, which require redox-driven surface reconstruction to reveal their intrinsic electrochemical activity.^13,25,76^ The α-Ni LH exhibited higher values of activation, followed by the NiAl LDH, and lastly, the β-Ni LH, according to the current density values achieved on each activation process (please note the scale values of the *y*-axis in Figure 1A for the β-Ni). In Figure 1B, the LSV curves show the good performance of the α-Ni LH compared to the other two phases when purified KOH is used. Although the lower activation values were shown for the β-Ni LH, it performed better than the NiAl LDH at higher potentials (Figure 1B), as expected from previous works.^13^

The overpotential values obtained at 10 mA/cm^2^ for each phase are 450 mV for the α-Ni LH, 486 mV for the β-Ni LH, and 535 mV for the NiAl LDH (see the inset in Figure 1B). It is important to note that, throughout this work, the OER performance is reported relative to the geometric area of the electrode, following common practice in the field. Alternative approaches, such as ECSA determination or quantification of active sites, are not straightforward and rely on assumptions. This normalization ensures consistency across the different LH phases studied and allows for meaningful comparison in the subsequent Fe incorporation experiments. This issue is currently a topic of significant discussion, as highlighted in several recent studies and reviews.^77–80^

These findings emphasize the distinct electrochemical behavior of these phases, particularly highlighting the favorable performance of the α-Ni LH sample in this context.^13^ The limited electrochemical performance of the NiAl LDH is attributed to the deliberate selection of aluminum (Al) as a nonactive cation within the LDH structure.

Once the incorporation processes were performed, ICP-MS analysis was carried out to quantify the Ni and Fe content in all samples and assess the effectiveness of the different approaches by comparing the Ni:Fe ratios. The as-synthesized samples exhibited high Ni:Fe ratios, approaching the detection limit of the instrument, due to the absence of Fe in these samples (Table 1). These values serve as a reference. Through the aging approach, the incorporation of Fe is observed in all three phases, resulting in a Ni:Fe ratio of approximately 900 in all cases, which indicates a consistent incorporation of Fe, regardless of the specific phase of the LH. For the electrochemical incorporation process, a significant increase in Fe incorporated was observed, leading to a decrease in the Ni:Fe ratio. Among the samples, the expanded ones exhibit the highest affinity to allocate iron, with the α-Ni LH showing the lower ratios, followed by the NiAl LDH and the β-Ni LH. To assess the effect of aging incorporation on Ni-based samples, various characterizations were performed, as detailed in SI Section 1. However, due to the significantly higher iron incorporation achieved through the electrochemical method, from now on, the discussion and analysis will primarily focus on this approach.

**Table 1:** Ni:Fe Ratios Calculated by ICP-MS for the Incorporation Approaches

	Ni:Fe ratio		
	no treatment	aging	electrochemical
α-Ni LH	17391	916	23.6
β-Ni LH	13780	934	57.5
NiAl LDH	12978	905	42.4

The strategy for electrochemically incorporating Fe from impurities in KOH involves an in situ approach, where cyclic voltammetries were performed over a range of activation potentials below typical Oxygen Evolution Reaction (OER) values. These experiments were carried out for up to 2000 cycles also using impure KOH 1 M (85%), as illustrated in Scheme 1B, on carbon paper electrodes (see the Experimental Section for more details). During the activation process, it is expected that the redox processes are involved, and the diffusion of the electrolyte through the sample allows that some of the impurities present in the KOH get attached to the layered structure, incorporating Fe into the Ni-based LHs. Figure 2 describes the CVs of the electrochemical incorporation approach for the three phases, where the successive cycles move from green to red color.

**Figure 2:**
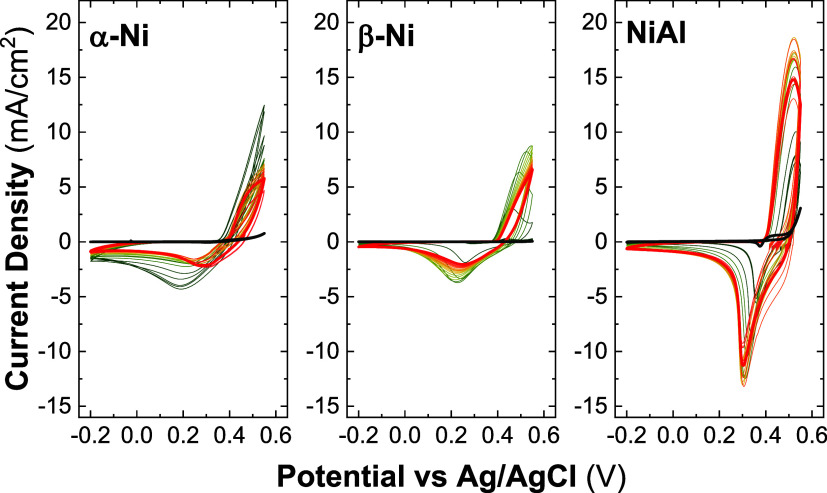
Voltamperograms for the electrochemical incorporation approach in a range of −0.2 to 0.55 V, in nonpurified 1 M KOH for samples α-Ni LH, β-Ni LH, and NiAl LDH over 2000 cycles. Greenish cycles correspond to the first ones, while reddish ones belong to the last ones.

Herein, the performance for the α-Ni LH initially improved up to the 300th cycle. From this point, the material remained stable up to the 1300th cycle when a decrease in the activity started to appear. In contrast, the β-Ni LH phase exhibited a constant enhancement in its performance along the activation cycles up to the 800th cycle, when it seemed to remain stable without significant changes. Notably, when looking at the last cycles, both α-Ni and β-Ni LHs showed a similar electrochemical behavior, suggesting a potential transformation where these two phases converge. As it happened in the aging treatment, a phase transformation that could be explained by the Bode diagram is observed, due to the displacement of the peaks from 2-θ = 8 ° to ca. 20 ° of the α-Ni LH, resembling the crystallographic fingerprint of a β-Ni LH after the aging in basic media.^81^

However, in this case, the transformation is forced by an electrochemical process, suggesting that both the α- and β-phases evolve into the Ni (oxy)­hydroxide phase –NiOOH– after the exposure to the cycles, as it was reported in previous works.^82^ It is important to note that the Ni (oxy)­hydroxide phase can be reached by both α-Ni and β-Ni LHs. However, the α-Ni LH reaches the NiOOH phase faster than the β-Ni LH, which requires long-duration electrochemical processes.^13^

Conversely, in the case of NiAl LDH, a significant and continuous increment in electrochemical activity is shown with increasing the number of cycles up to the 1200th cycle. This phase exhibits higher activity values compared to the as-synthesized phase when activated in purified KOH. The enhancement in electrochemical performance observed for this phase during the electrochemical incorporation process can be related to two main processes. The first one is related to the dissolution of aluminum (leaching process) from the hydroxide layer into the alkaline media due to the potential stress the material is forced to, as was reported previously for different aluminum phases in KOH electrolytes.^16,83–85^ The second one deals with the incorporation of Fe in the vacancies left by the Al that led to the formation of a highly OER active NiFe-like phase (vide infra). This leaching of amphoteric metals such as Zn or Al in LDHs is a well-studied approach for generating vacancies within the layered structure, allowing for the incorporation of additional cations or modification of the electronic environments of the cations in the layer.^16,86–89^ This process is typically applied to samples containing electroactive cations, such as Co, Ni, or Fe, with a low percentage of Zn or Al in the structure. To enhance Al leaching in these processes, higher KOH concentrations and higher temperatures are commonly used.

Once the electrochemical Fe incorporation approach was completed, the treated electrodes (hydroxides over carbon paper) were measured in purified KOH to observe and compare their electrochemical properties, as was done before for aged and pristine materials. In Figure 3, the activation CVs and the comparison between the LSV are shown for the electrochemically treated LHs. In the activation CVs for the three phases (see Figure 3A), there are no significant changes in the intensity values between the initial cycle (depicted in black) and the subsequent cycles (color-coded according to the sample). This is attributed to the fact that the material had already been activated through the electrochemical Fe incorporation method, and no further activation occurred after the treatment applied to the different phases of Ni-based LHs.

**Figure 3:**
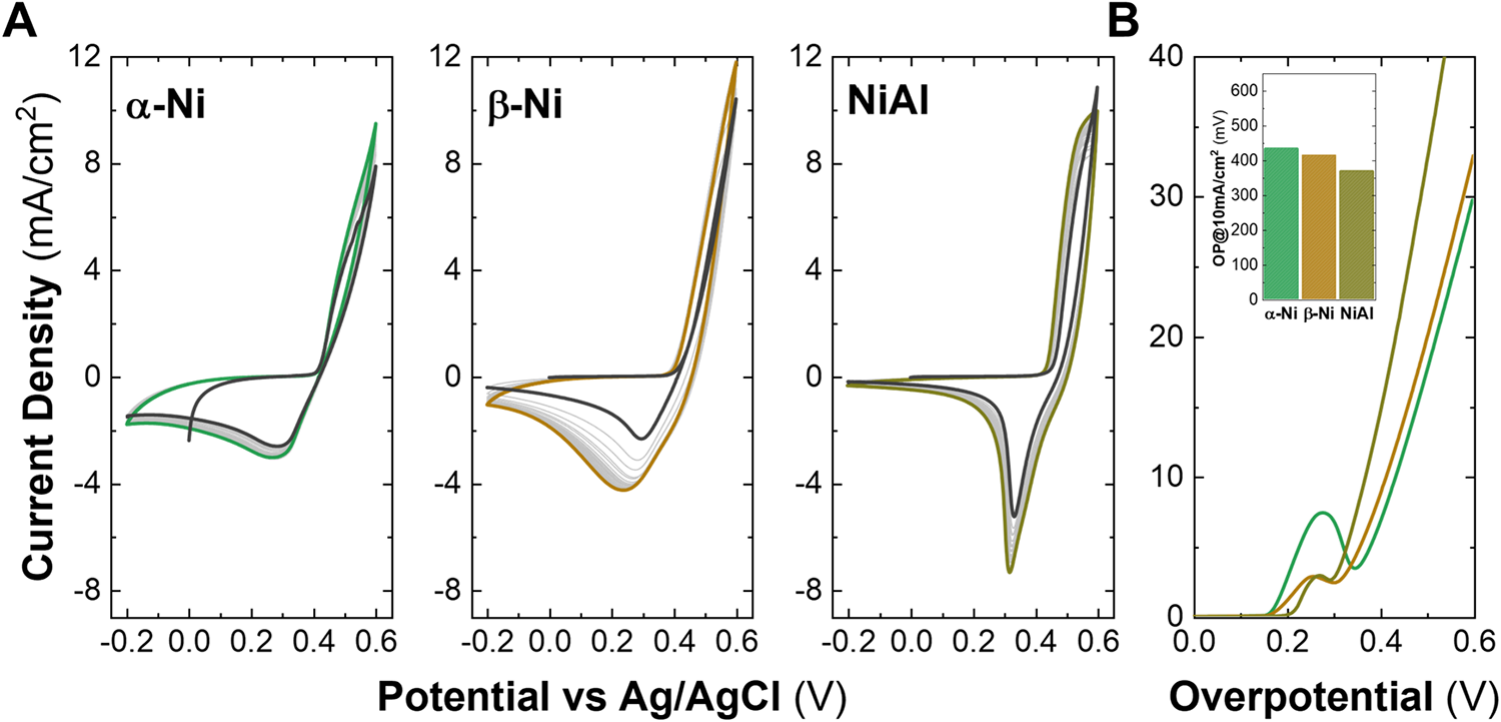
Electrochemical characterization in purified 1 M KOH of the samples treated by electrochemical incorporation: Cyclic voltammetries for the samples α-Ni LH, β-Ni LH, and NiAl (A). Linear sweep voltammetry curves measured at 5 mV/s (B) and overpotential values required for a current density of 10 mA/cm^2^ (inset in B).

LSVs of activated electrodes after electrochemical incorporation in Figure 3B revealed a change in the electrochemical performance trend shown for both the as-synthesized and aged electrodes described previously. Here, the α-Ni LH presents the highest values of OP@10 mA/cm^2^, followed by the β-Ni LH, with the NiAl LDH having the lowest OP values. The OP values at 10 mA/cm^2^ obtained are as follows (inset of Figure 3B): 435 mV for the α-phase, 415 mV for the β-phase, and 370 mV for the LDH phase. Typical OP values of on-purpose-synthesized NiFe LDHs ranged between 250 and 350 mV at 10 mA/cm^2^.^17,20,90–93^ A comparison between the LSVs and the values of OP@10 mA/cm^2^ obtained by the electrochemical measurements in purified KOH for the nontreated and the treated samples with 1 M nonpurified KOH is presented in Figure S5A,B, respectively.

The electrochemical Fe incorporation was also conducted in 6 M KOH 85% (see Figure S6), and the results obtained after the characterization in purified KOH (Figure S7A,B) exhibited similar trends. The NiAl LDH displayed the most significant enhancement in terms of OP@10 mA/cm^2^, followed by the α-Ni LHs and finally the β-Ni, which presents a lower value but still better than its pristine value. The slight deviations observed may be attributed to the decomposition of the electrode caused by the high concentration of KOH and the prolonged duration of the process. A comparison of the LSVs obtained for both electrochemical incorporations at 1 and 6 M is shown in Figure S7C.

The improved performance of the NiAl LDH phase after the electrochemical process can be ascribed to the framework Fe incorporation in the structure of the LDH due to the leaching of structural Al, establishing Fe-rich regions within the NiAl LDH layers. It is worth highlighting that although the electrochemically treated NiAl LDH phase that incorporates iron has an interesting electrochemical performance, the values are far from the typical ones shown by purposely synthesized NiFe LDH (see Figure S9). Based on ICP-MS data (Table 1), it is worth highlighting that the NiAl LDH after the electrochemical treatment exhibits a ratio notably distant from the typical ratios observed in NiFe LDHs, which typically range between 2 and 4.

This electrochemical Fe incorporation method was also tested on NiFe-LDHs using nonpurified KOH at 1 and 6 M (See Figure 4A). For the sample incorporated in 1 M KOH, as the number of cycles increases (from green to red), a slight decrease in current intensity is observed, most notably in the reduction peak, which exhibits a variation of approximately 1 mA/cm^2^. When analyzing the activation CVs and LSV of the sample, we first observe that the CV becomes activated. At 6 M, the behavior remains consistent; however, a small plateau appears before the reduction peak during the activation cycles, which becomes more pronounced as the cycles progress. After electrochemical characterization in purified 1 M KOH, this plateau disappeared, and both samples exhibit similar behavior.

**Figure 4:**
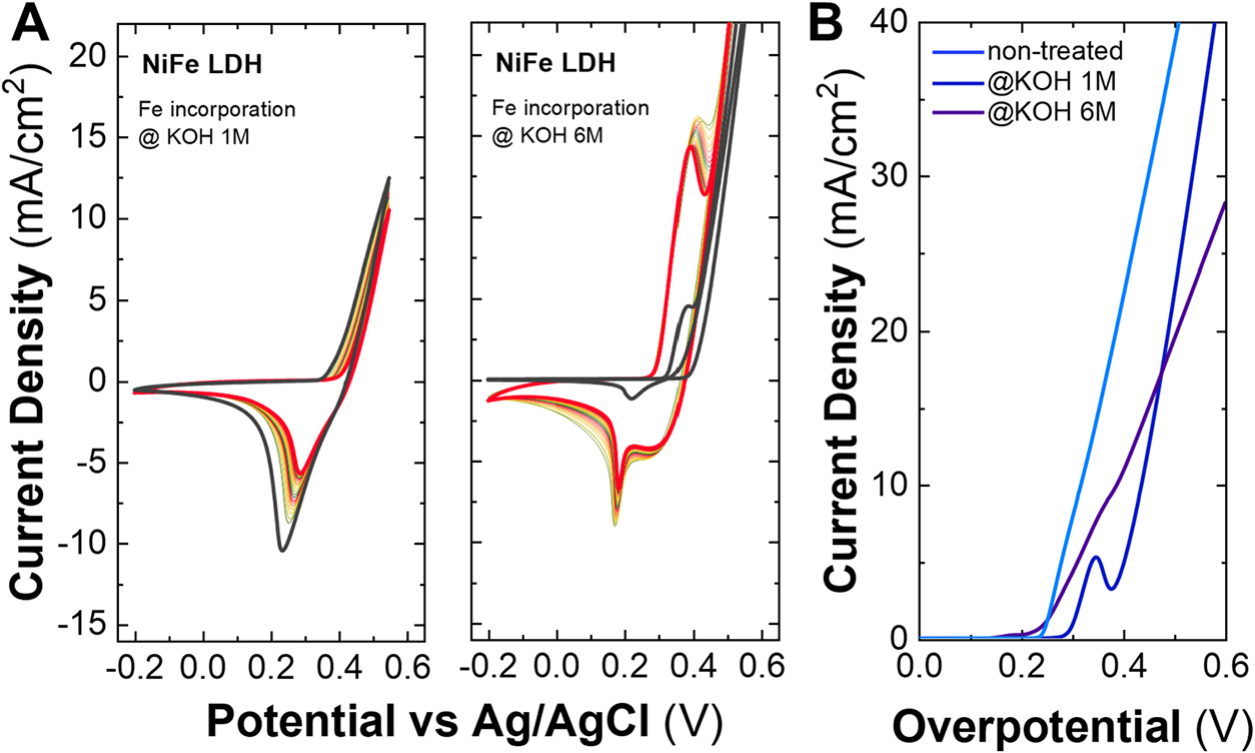
Voltamperograms for the electrochemical incorporation approach in a range of −0.2 to 0.55 V, in nonpurified 1 M KOH for NiFe LDH over 2000 cycles. Greenish cycles correspond to the first ones, while reddish ones belong to the last ones (A). Electrochemical characterization in purified 1 M KOH of the sample treated by electrochemical incorporation: Linear sweep voltammetry curves were measured at 5 mV/s (B).

In both cases, when measuring the LSV (Figure 4B), the OP@10 mA/cm^2^ observed for both samples treated at 1 and 6 M decreases in comparison with the bare sample, being 390 and 430 mA/cm^2^. Mass loading control before and after the electrochemical measurements confirms that there is no significant change in the amount of material in the electrode at the end of the experiments, highlighting the effectiveness on the spray coating deposition method. The decrease in NiFe-LDH performance may be attributed to inherent and well-documented stability issues of these materials in alkaline media during OER processes. These include iron dissolution and reincorporation, which can lead to the formation of less reactive metal oxide layers, as well as structural reconstruction and chemical degradation.^80,94–97^ This demonstrates that these Fe incorporation treatments, at least under the conditions used in this work, are not an efficient strategy for improving the electrochemical performance for pure NiFe LDH samples.

Scanning electron microscopy with energy-dispersive X-ray spectroscopy (SEM-EDX) analysis was performed for the electrodes deposited on carbon paper both before and after electrochemical treatment in nonpurified 1 M KOH (see Figures 5 and SI10–12). Regarding the morphology, no appreciable change was observed between the samples attached to the carbon paper. For the EDX analysis, in all the cases, the pretreated samples displayed trace amounts of Fe, ascribed to the initial number of metal signals observed in the carbon paper without any electroactive material (Figure S9). All the samples exhibit an increase in the Fe signals after the electrochemical incorporation process, as can be observed in Figures S10, S11, and S12, for the α-LHs, β-LHs, and LDH, respectively. Table S1 summarizes the EDX results, showing that the Fe content in the post-treatment samples is generally between 0.5 and 0.7%.

**Figure 5:**
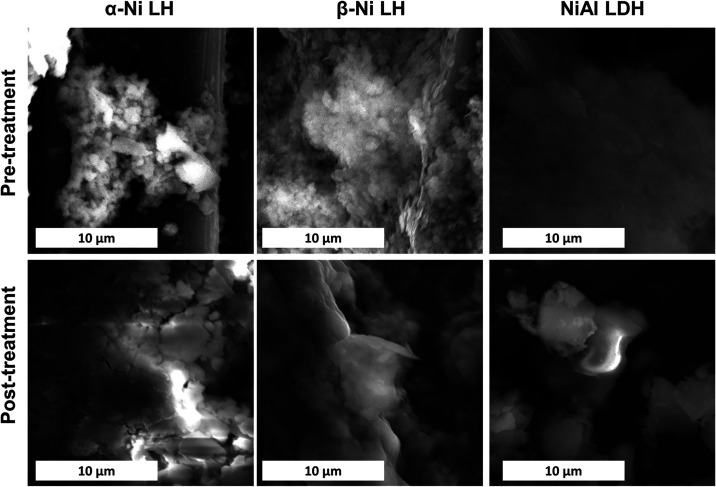
SEM images of the Ni-based LHs supported on carbon paper, before and after treatment by electrochemical methods.

To further investigate the morphology of the particles without interference from the electrode, samples were collected from the electrodes both before and after the electrochemical process. This was achieved by scraping the catalyst layer, which was then dispersed in ethanol to prepare inks for transmission electron microscopy (TEM) analysis. Additionally, the dispersion was deposited onto a silicon (Si) substrate for SEM-EDX measurements. These analyses were conducted to confirm that the observed particles correspond to Ni-based LHs rather than to species originating from the current collector or the Si substrate. The resulting images are presented in Figure 6.

**Figure 6:**
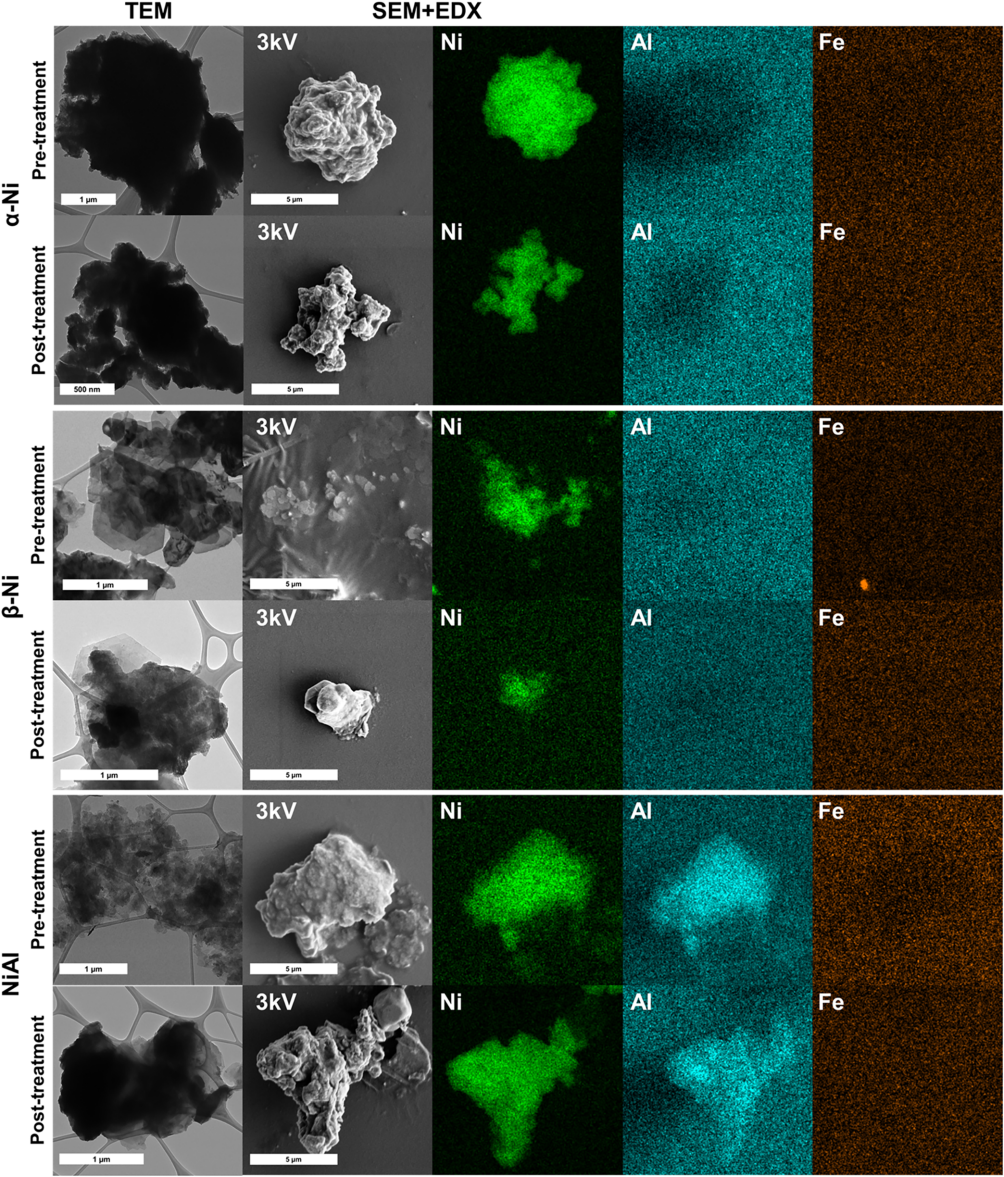
TEM and SEM-EDX images of the Ni-based LHs recovered from the electrodes before and after the electrochemical treatment. SEM images were taken at 3 kV.

In the α-LH phase, increased particle agglomeration is observed, and its morphology appears to be flatter compared to the typical flower-like structure characteristic of this phase.^13,98^ In the β-phase, a slight degradation of the edges after the electrochemical incorporation is observed by the SEM image; however, TEM analysis reveals that some particles remain with the typical hexagonal morphology of these samples, despite the electrochemical stress. For the NiAl phase, the agglomeration of the particles is also observed, comparing the sample before and after the treatment. Before incorporation, small groups of particles are observed; although aggregated, they are somewhat dispersed, while after incorporation, it is observed how much larger, more compact particles are formed. In the case of the NiFe LDHs (Figure S13), there are no appreciable morphological changes despite a better form at the edges of the sample after the electrochemical treatment. Figure S14 shows the EDX spectra corresponding to the elemental mapping presented in Figure 6. A strong aluminum signal is observed in all spectra (also for samples that do not contain Al), although the elemental maps do not display a clear distribution of this element. This aluminum signal is most likely an artifact, attributable to external factors such as contributions from the sample holder or the sputter-coating process, rather than to the intrinsic composition of the samples.

ICP-MS analysis was done on the electrodes prior to and after the electrochemical incorporation treatment, to determine the content of Ni, Fe, and Al of the samples, to gain insights into the atomic composition, and to compare with the previously explored EDX (Table 2). In all the samples (except in the NiFe LDH), the Fe content increases as expected, observing similar percentages to those obtained in EDX. For the α-Ni LH, a noticeable increase in Aluminum content was also observed. This may be due to adsorption from the glass container used during the experiments; however, this large increase of Al content was only in this sample. In the case of NiAl LDH, an important decrease in the Al content is observed, going from ca. 40% to lower than 18%, confirming the leaching of the aluminum during the electrochemical process.

**Table 2:** Ni, Fe, and Al Atomic Percentages Obtained by ICP-MS for the Electrochemical Incorporation Approach for the Ni-Based LHs

sample	Ni (%)	Fe (%)	Al (%)
α-Ni LH Pre	99.16	0.08	0.76
α-Ni LH Post	92.42	0.59	7.00
β-Ni LH Pre	98.93	0.09	0.99
β-Ni LH Post	99.01	0.25	0.75
NiAl LDH Pre	57.01	0.26	42.73
NiAl LDH Post	81.26	1.21	17.52
NiFe LDH Pre	65.81	33.55	0.64
NiFe LDH Post	65.16	32.39	2.45

In the case of NiFe LDHs, although electrochemical performance issues were observed after Fe incorporation treatment, the Ni and Fe contents remained stable. This suggests, on one hand, that no additional iron was successfully incorporated during the experiment—remarking that this electrochemical incorporation method is not suitable for as-synthesized NiFe LDHs, and, on the other hand, that redissolution processes may be occurring in the system due to prolonged cycling, where the material undergoes both oxidative and reductive processes.

Once the morphology and the composition of the samples were explored, post-mortem analysis using PXRD was conducted on the phases after the electrochemical approach to examine potential changes in their crystalline structures. Figure S15 depicts the diffractograms of the samples deposited on carbon, both before and after the treatment, with a focus on the primary peaks below 2-θ = 25°. In the case of α-Ni LH, a notable shift in the primary reflection is evident. In the as-synthesized sample, the 003 reflection is positioned below 2-θ = 10°. However, in the post-treatment sample, this peak shifts to around 2-θ = 13°. This shift was observed in previous works, highlighting the presence of the Ni-(oxy)­hydroxide phase.^13^ For the β-Ni LH, the as-synthesized sample displays the typical 001 reflection close to 2-θ = 19°. However, after treatment, the peak around 19° remains with less intensity, and a new peak near 13° emerges. This confirms the transformation of β-Ni LH into a Ni (oxy)­hydroxide phase, resembling the behavior of the treated α-Ni LH, as previously seen in the CVs, where both α-Ni and β-Ni LHs end into the (oxy)­hydroxide. Regarding the NiAl LDH sample, the peak observed in the pristine sample above 2-θ = 11° persists but appears less crystalline after treatment. This reduction in crystallinity could be attributed to the presence of new amorphous phases related to the incorporation of iron in the material and also to the leaching of Al from the LDH structure.

Raman spectroscopy is a powerful technique that allows the detection of different vibrational modes in transition metals due to the high sensitivity of the technique. In the case of α-Ni and β-Ni LHs, it has been proven that after an electrochemical treatment in purified KOH, the samples end up in the NiOOH phase, due to the characteristic vibrations ca. 470 and 550 cm^–1^.^99–104^ The NiOOH can be either β-NiOOH or γ-NiOOH, with the β-phase being a well-defined structure and the γ-phase being a collection of different crystalline structures. Typically, the phase obtained during the electrochemical processes is γ-NiOOH due to the overcharge. Due to the lack of certainty in the literature to differentiate them, the oxyhydroxide phase formed in situ and observed by ex situ experiments will be noted simply as NiOOH.

Figure 7 depicts the Raman spectra of the samples before and after electrochemical incorporation and electrochemical characterizations in the 100–1000 cm^–1^ range. In the case of pristine α-Ni, a peak located ca. 455 cm^–1^ is observed, which corresponds to the typical vibrations of the α-Ni–OH lattice.^104,105^ After treatment and electrochemical characterizations, peaks at 477 and 556 cm^–1^ appeared, corresponding to the NiOOH phase. As well for β-Ni LHs, in the nontreated sample bands at 313 and 448 cm^–1^ are observed, which are related to the Eg β-NiOH and A1g β-NiOH vibrations.^104,105^ Once the incorporation and the electrochemical performance were done, new bands at 478 and 557 cm^–1^, confirmed the presence of the NiOOH phase.

**Figure 7:**
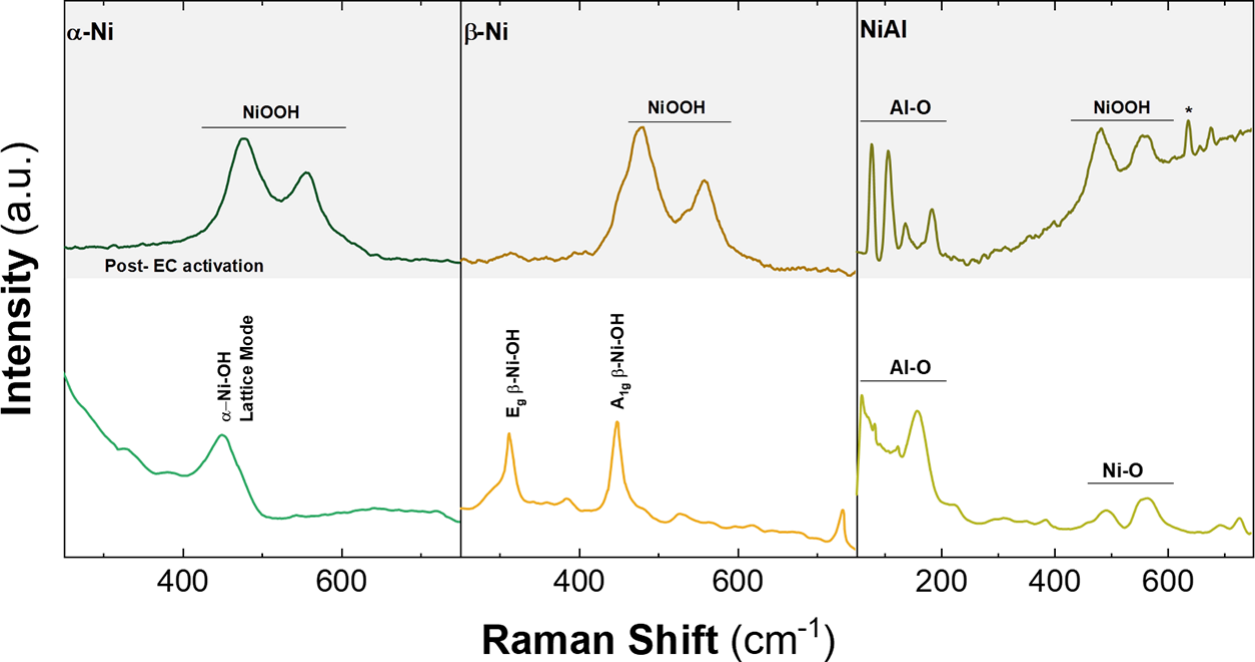
Raman spectra of the Ni-based LHs deposited in carbon paper, before and after the electrochemical incorporation approach; α-LH (Left), β-LH (center), and NiAl-LDH (right). The asterisk highlights the presence of a band related to the vibrational modes of FeOOH.

The pristine NiAl LDH sample exhibits bands below 200 cm^–1^ characteristic of the hydrotalcite-like structure^106^ as well as some bands in the range of 500 and 600 cm^–1^ related to M-O vibrations. After the treatment and characterization, the presence of NiOOH bands is observed at 480 and 558 cm^–1^. This differs from the behavior of the long-term performance of a NiAl in the purified electrolyte, which is nonreactive and does not form any other electroactive phase.^13^ The presence of the (oxy)­hydroxide suggests the formation of a new phase due to Fe incorporation. Along this front, a peak over 638 cm^–1^—highlighted by an asterisk—is observed, related to the vibrational modes of FeOOH,^100^ suggesting that the Fe was incorporated in the layer. The changes under 200 cm^–1^ correspond to modifications in the Al–O vibrations resulting from the leaching of this metal. In general, the presence of the NiOOH phase is observed in all cases after the electrochemical performance tests, and a shift in the position values of the characteristic peaks is noted compared to those observed in the literature (Figure S16). This can be related to the incorporation of Fe on the materials: the variation in the Raman shift of NiOOH vibrations toward higher values is dependent on the amount of incorporated Fe.^102,107^

X-ray photoelectron spectroscopy (XPS) in Al Kα radiation was performed on the electrodes prior to and after the electrochemical treatment to gain further information about the chemical environment of the cations studied, as presented in Figure 8. Ni^II^ typically exhibits characteristic peaks ca. 855.8 eV (Ni 2p_3/2_) and 873.3 eV (Ni 2p_1/2_), along with their respective satellite features close to 861.5 and 879.4 eV.^108^ This spectral behavior is followed in all samples prior to treatment (see Figure 8A). After the electrochemical Fe incorporation approach, the overall spectral features remain consistent, indicating no major alterations in the Ni chemical environment. However, a shift in the Ni 2p peaks is evident for both the α-Ni and NiAl samples. In the case of α-Ni, the peaks shift toward lower binding energy values (approximately −1 eV), while in the NiAl-LDH sample, a positive shift of around +1 eV is observed. These shifts are likely attributable to charging effects, as the measurements were conducted directly on the carbon paper substrate used during the electrochemical treatment. It is also important to note that although the shifts in α-Ni and NiAl are the most apparent, all samples exhibit a slight deviation from the nominal 855.8 eV position typically associated with the Ni 2p_3/2_ main peak. On the other hand, Fe^III^ typically presents peaks at 711.8 and 723.6 eV, corresponding to the Fe 2p_3/2_ and Fe 2p_1/2_ signals, respectively.^108^

**Figure 8:**
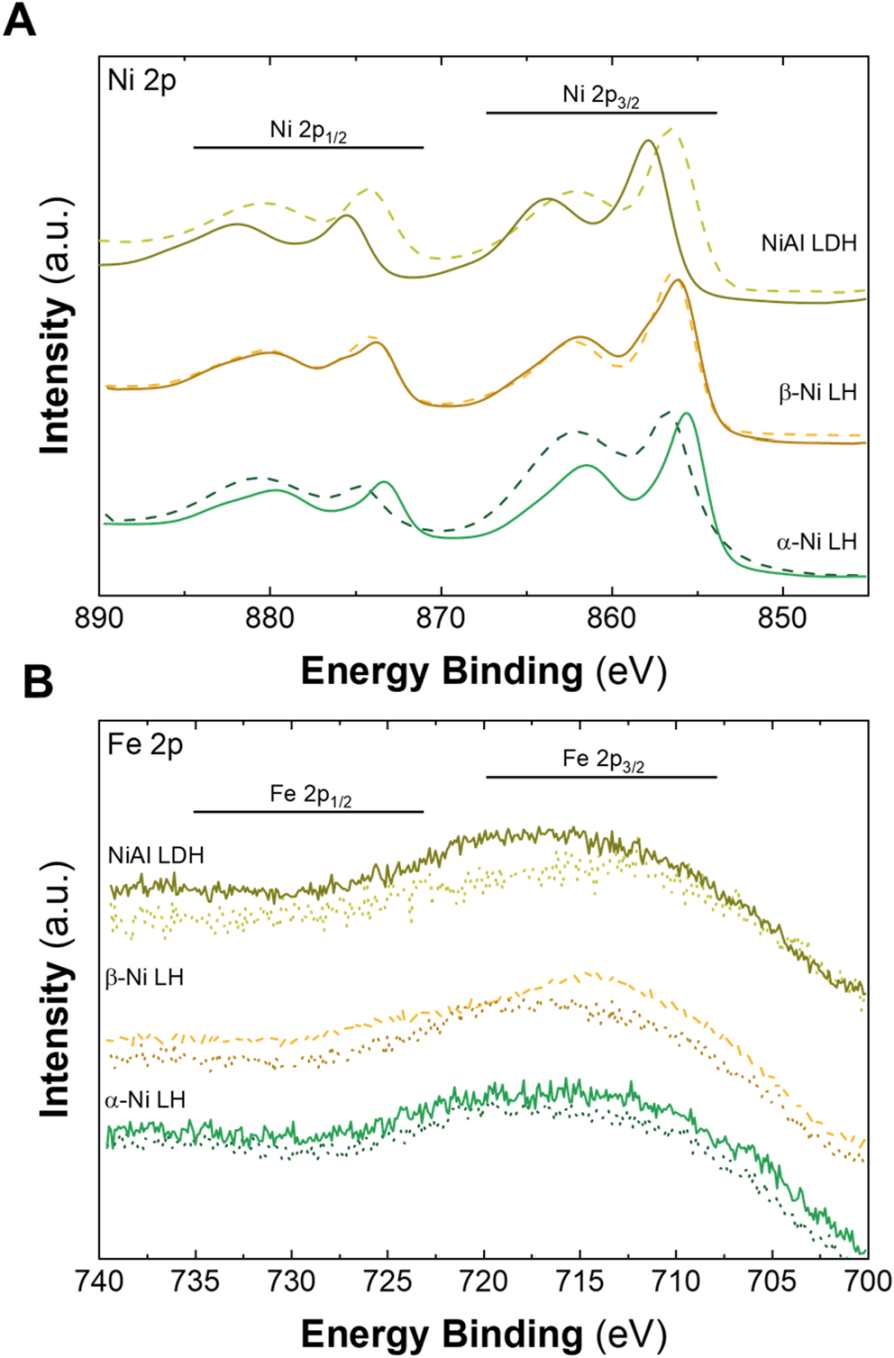
XPS of the Ni-based LHs deposited in carbon paper before and after the electrochemical incorporation approach Ni 2p (A) and Fe 2p (B).

In the case of the Ni-based LH samples, the Fe signal is poorly defined (Figure 8B), and no distinct peaks can be identified. This is expected for the samples prior to the Fe incorporation. However, in those subjected to electrochemical Fe incorporation, a detectable Fe signal was expected. The weak or absent Fe features in the post-treatment samples stand in contrast to those observed in intentionally synthesized NiFe-LDHs (see Figure S17). Since the Fe 2p peaks overlap with the Ni LMM Auger—leading to possible interferences when the Fe content is low, XPS using Mg Kα radiation was also performed for the Ni-based LHs electrodes, as presented in Figure S18. The resulting Fe 2p signals were weak and could not be reliably deconvoluted, likely due to the high surface sensitivity associated with this lower-energy radiation as well as the presence of intense F 1s peaks near the Fe 2p region, attributed to the Nafion binder used in the electrode preparation (see Figure S19). These discrepancies observed in both Kα radiations arise from the surface-sensitive nature of the XPS technique, in combination with the low amount of Fe incorporated into the material, observed by other quantification techniques.

To further investigate the incorporation of Fe into these materials, X-ray absorption (XAS) measurements were performed in both the near-edge region (XANES) and the extended region of the spectrum (EXAFS). Given the chemical selectivity of this technique, it is possible to analyze the average electronic and structural characteristics of the Ni atoms as well as the incorporated Fe. Figure 9A presents the XANES spectra measured at the Ni K-edge for the electrodes before electrochemical incorporation (dashed lines) and those recovered after the process (solid lines). It can be observed that the treatment of the materials does not affect the position of the absorption edge (vertical dashed line at 8342 eV), indicating that the oxidation state of Ni remains at Ni^II^.^13^ Since this is an ex situ process, it is worth mentioning that the electrodes were not exposed to electrochemical stimuli, making the reversibility of Ni phases to Ni^II^ expected.

**Figure 9:**
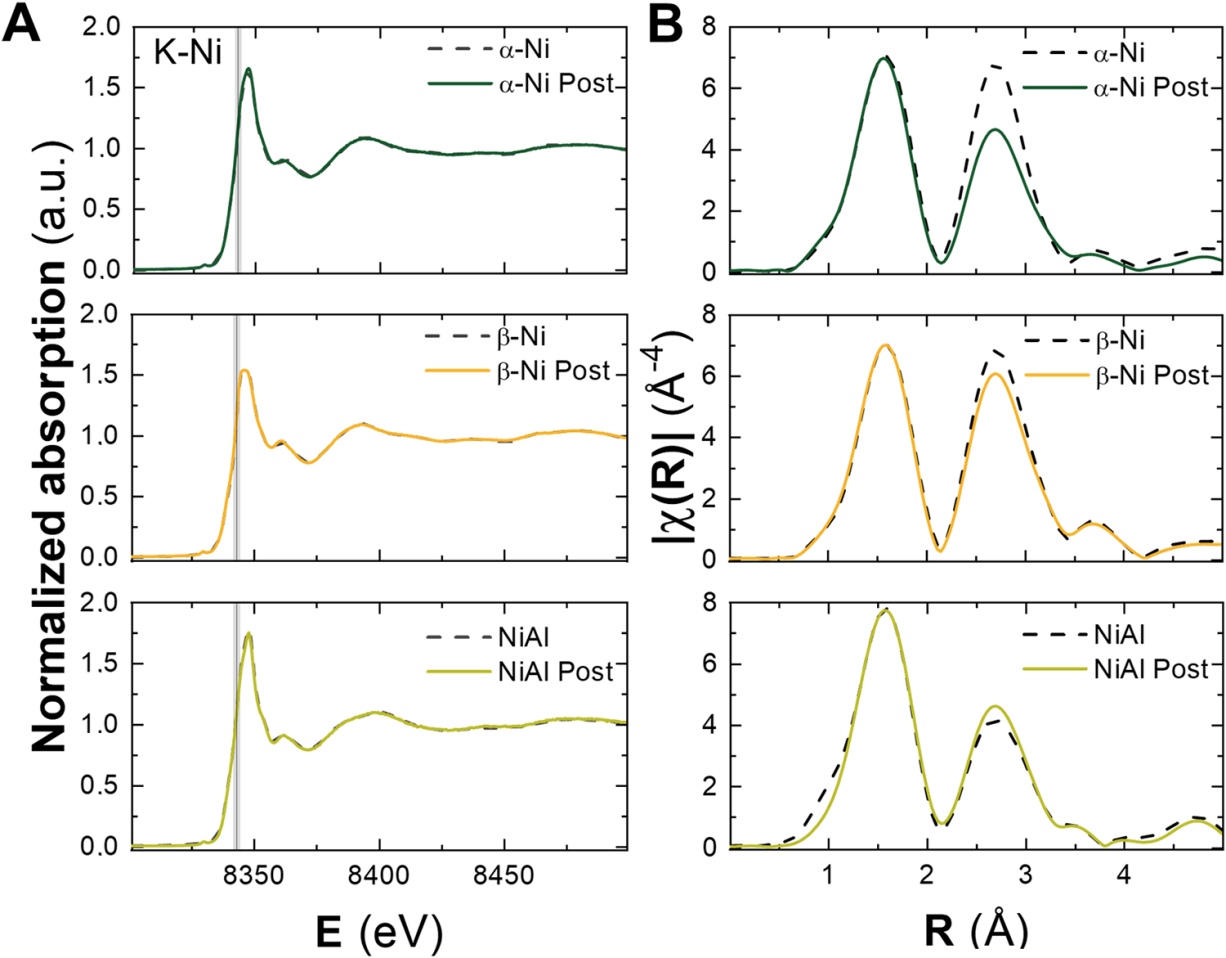
XANES spectra (A) and EXAFS data (B) for the Ni-based LHs measured at the Ni K-edge, before (dashed line) and after (solid line) electrochemical treatment.

On the other hand, structural information regarding the local environment of Ni atoms can be obtained from the analysis of the EXAFS region of the absorption spectrum. The Fourier transforms of the EXAFS oscillations are shown in Figure 9B. In all cases, two main peaks are observed, corresponding to the first two coordination shells of Ni. The first peak, located at 1.5 Å (without phase correction), corresponds to the nearest oxygen neighbors of Ni, which remains identical between samples before and after EC experiments. On the other hand, the second peak is associated with the next-nearest neighbors of Ni. In the pristine α-Ni and β-Ni samples, these next-nearest neighbors are exclusively Ni atoms, whereas in the NiAl sample, they consist of both Ni and Al atoms.

Notably, changes in the amplitude of this peak are observed upon comparison of treated and untreated samples, which could be linked to variations in the coordination number of this second coordination shell. To quantify the effect of this change, structural parameter refinements were performed. As previously described, the fitting was based on a structural model where the first coordination shell around Ni atoms consists of oxygen atoms, while the second shell consists of Ni atoms (for the α-Ni and β-Ni samples) or a mixture of Ni and Al (for the NiAl sample). Figure S20 and Table S2 present the refined structural parameters obtained from the fitting. As expected, neither the coordination number nor the interatomic distance in the first coordination shell undergoes significant changes with Fe incorporation into any of the samples. However, modifications in the coordination number of the second-nearest Ni neighbors are observed. For the α-Ni and β-Ni samples, a reduction in this parameter is observed upon Fe incorporation, which could initially suggest that some Ni atoms have been substituted by Fe. However, given that the amount of Fe incorporated is around 1%, it is not appropriate to conclude that this decrease is due to Ni substitution. A more plausible explanation is the generation of Ni vacancies after the samples are exposed to electrochemical treatment. For the NiAl sample, an increase in the amplitude of the second coordination shell contribution is observed. The fitting reveals that this can be attributed to a decrease in the Ni–Al coordination. This suggests that the treatment generated aluminum vacancies, which could subsequently be occupied by iron, although the incorporation is difficult to observe due to its low concentration.

As mentioned earlier, given the low Fe incorporation levels, it is challenging to detect changes by monitoring the Ni atoms. Therefore, taking advantage of the chemical selectivity of this technique, XAS measurements were also performed at the Fe K-edge. However, due to the low signal intensity, it was only possible to obtain XANES spectra, as the EXAFS region could not be analyzed with sufficient quality. Figure 10 presents the XANES spectra measured at the Fe K-edge for the studied samples along with those of carbon paper and commercial NiFe-LDH as a reference.

**Figure 10:**
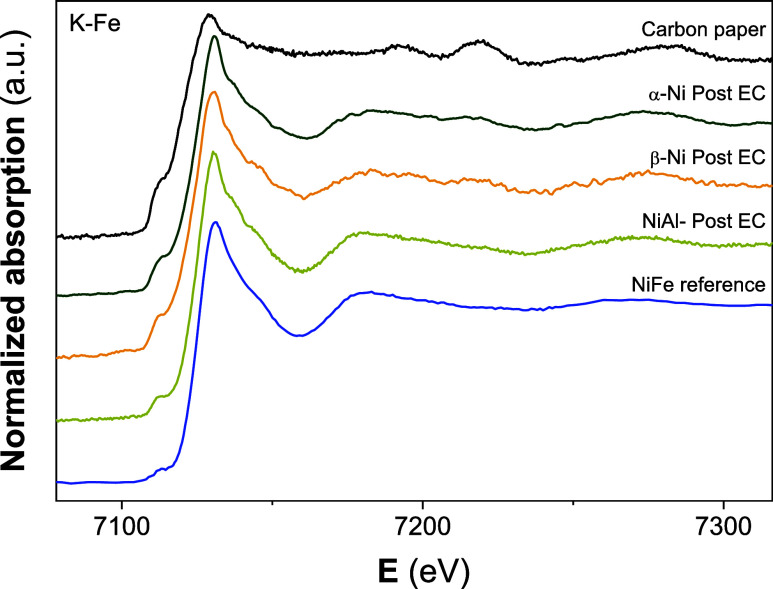
XANES spectra for the Ni-based LHs samples, along with those of carbon paper and NiFe reference samples, measured at the Fe K-edge.

Since EDX analysis revealed a slight Fe signal from the carbon paper used as the support, its XANES spectrum was also measured. As shown in Figure 10, the Fe signal from the support is markedly different from that of the Fe observed in the Ni samples after the electrochemical incorporation, indicating that the iron detected in the LHs originates from a different species than those present in the carbon paper and is also present in approximately 10-fold higher concentration. It is important to mention that the Fe signal from the carbon paper is identical to that obtained for the three Ni samples prior to electrochemical treatment, confirming that all the Fe detected in these samples, at that stage, originates exclusively from the support.

Additionally, a reference spectrum of a commercial NiFe-LDH where Fe is intrinsically incorporated into the structure is presented. The key spectral features of the studied samples closely match those of the reference sample spectrum, providing compelling evidence that the Fe is primarily incorporated into the Ni LHs lattice via the substitution of Ni and/or Al atoms. Based on these findings, it is suggested that the electrochemical incorporation of Fe occurs using commercial electrolytes with high impurity levels. However, due to the small amount of Fe incorporated, it is challenging to confirm the nature of Fe by analyzing its closer interactions. To explore the effect of different commercial references, the electrochemical incorporation was also evaluated using KOH with lower iron impurities (see Figures S21 and S22). Unlike previous results, there is no significant change in the CVs and no improvement in the overpotential values. This underscores the importance of the quantity of Fe impurities in enhancing the electrochemical performance. Future work should be done exploring electrolytes intentionally enriched with higher Fe content.

A comparison between the incorporation processes using KOH #1 (the KOH used for the electrochemical incorporation experiments described until now) and KOH #2 (A KOH with lower iron impurities) is shown in Figure S23, where the differences between the processes are more clearly observed. It is important to note that even using KOH #2 as electrolyte, the results are still slightly better than those obtained through Fe incorporation by aging using KOH #1, as shown in Figure S24. This confirms that electrochemical approaches are more efficient than aging methods for Fe incorporation, regardless of the KOH employed.

In general, an improvement is observed due to the Fe incorporation through electrochemical approaches, highlighting that these impurities can enhance the electrochemical behavior under studied conditions. However, the differences by using electrolytes with different quantities of impurities suggest that the improvement strongly depends on multiple factors, including the reagent supplier, impurity level, material deposition, and incorporation methods. This study emphasizes the importance of analyzing the electrolyte in any electrochemical study and the importance of the impurities (chemical nature and concentration).

Therefore, to conduct a systematic study and achieve the goal of identifying new in situ phases through metal incorporation by electrochemical methods, and properly decipher how the incorporation occurs, it is crucial to control the number of variables, optimize the electrochemical incorporation parameters—such as electrochemical technique, current, potential and time—and to use known cation concentrations, in line with some recently published works.^109,110^

## Conclusion

This study investigates the impact of Fe impurity incorporation on nickel-based layered hydroxides when used as electrocatalysts for the OER in alkaline water electrolysis. For this, two distinct Fe incorporation methods have been explored: a standard KOH purification process and an electrochemical activation approach. The results show that electrochemical activation is more effective, with expanded LH phases exhibiting a higher affinity for Fe, resulting in improved electrochemical performance. Prolonged electrochemical activation under nonpurified KOH led to the transformation of both β-Ni and α-Ni LHs into a Ni (oxy)­hydroxide phase. The α-Ni LH reached the Ni oxyhydroxide phase more easily, while β-Ni required long-term cycles to transform. For both phases, this transformation resulted in an improved electrochemical performance. In the case of the NiAl phase, aluminum was lost into the alkaline medium due to a leaching effect, leading to vacancies that favored the Fe incorporation in the NiAl LDH, suggested by both Ni and Fe (oxy)­hydroxide peaks in Raman spectroscopy. Moreover, synchrotron-based techniques confirm the presence of Fe in the layers, without affecting the electronic environment of the nickel and suggesting possible structure incorporation of Fe due to the similarity in the XANES spectra of Fe in the Ni-based LHs and a commercial NiFe LDH.

This in situ treatment created phases that enhance the electrochemical performances of the bare NiAl-LDH. However, the obtained Ni:Fe ratios were smaller than those characteristic for ex situ synthesized NiFe-LDHs, which results in lower OER performance compared to these on-purpose-synthesized materials, including commercial ones. Interestingly, for pure NiFe-LDHs, these types of in situ treatments or doping methods were found to be ineffective, indicating that such strategies are not suitable for already optimized NiFe-based catalysts.

Despite the improvements observed through electrochemical iron incorporation, the reproducibility of this transformation strongly depends on the amount of impurities in the electrolyte used as the source of Fe ions. This makes the process somewhat unpredictable when solely relying on electrolyte impurities in realistic devices. This emphasizes the importance of systematically studying electrolyte impurities (whether they originate from KOH or materials used in electrolyzers, such as pipes, gaskets, etc.) and underscores the need for controlling Fe content when utilizing this incorporation method.

## Supplementary Material



## Notes

Experimental details: XRD, ICP-MS, SEM-EDX, XPS, Raman, and electrochemical measurements; Fe incorporation (PDF)

## References

[1] Chu S., Majumdar A. (2012). Opportunities and Challenges for a Sustainable Energy
Future. Nature.

[2] Yan Z., Hitt J. L., Turner J. A., Mallouk T. E. (2020). Renewable Electricity
Storage Using Electrolysis. Proc. Natl. Acad.
Sci. U.S.A..

[3] Lagadec M. F., Grimaud A. (2020). Water Electrolysers with Closed and Open Electrochemical
Systems. Nat. Mater..

[4] Zhang Y., Ouyang B., Xu J., Jia G., Chen S., Rawat R. S., Fan H. J. (2016). Rapid Synthesis
of Cobalt Nitride
Nanowires: Highly Efficient and Low-Cost Catalysts for Oxygen Evolution. Angew. Chem..

[5] Dionigi F., Zeng Z., Sinev I., Merzdorf T., Deshpande S., Lopez M. B., Kunze S., Zegkinoglou I., Sarodnik H., Fan D., Bergmann A., Drnec J., de Araujo J. F., Gliech M., Teschner D., Zhu J., Li W.-X., Greeley J., Cuenya B. R., Strasser P. (2020). In-Situ Structure
and Catalytic Mechanism of NiFe and CoFe Layered Double Hydroxides
during Oxygen Evolution. Nat. Commun..

[6] Dionigi F., Zhu J., Zeng Z., Merzdorf T., Sarodnik H., Gliech M., Pan L., Li W., Greeley J., Strasser P. (2021). Intrinsic Electrocatalytic
Activity for Oxygen Evolution of Crystalline 3d-Transition Metal Layered
Double Hydroxides. Angew. Chem., Int. Ed..

[7] Diaz-Morales O., Ledezma-Yanez I., Koper M. T. M., Calle-Vallejo F. (2015). Guidelines
for the Rational Design of Ni-Based Double Hydroxide Electrocatalysts
for the Oxygen Evolution Reaction. ACS Catal..

[8] Tareen A. K., Priyanga G. S., Khan K., Pervaiz E., Thomas T., Yang M. (2019). Nickel-Based Transition Metal Nitride Electrocatalysts for the Oxygen
Evolution Reaction. ChemSusChem.

[9] Yang L., Liu Z., Zhu S., Feng L., Xing W. (2021). Ni-Based Layered Double
Hydroxide Catalysts for Oxygen Evolution Reaction. Mater. Today Phys..

[10] Zhou D., Li P., Lin X., McKinley A., Kuang Y., Liu W., Lin W.-F., Sun X., Duan X. (2021). Layered Double Hydroxide-Based
Electrocatalysts for the Oxygen Evolution Reaction: Identification
and Tailoring of Active Sites, and Superaerophobic Nanoarray Electrode
Assembly. Chem. Soc. Rev..

[11] Hager L., Hegelheimer M., Stonawski J., Freiberg A. T. S., Jaramillo-Hernández C., Abellán G., Hutzler A., Böhm T., Thiele S., Kerres J. (2023). Novel Side Chain Functionalized Polystyrene/O-PBI
Blends with High Alkaline Stability for Anion Exchange Membrane Water
Electrolysis (AEMWE). J. Mater. Chem. A.

[12] Sanchis-Gual R., Hunt D., Jaramillo-Hernández C., Seijas-Da
Silva A., Mizrahi M., Marini C., Oestreicher V., Abellán G. (2023). Crystallographic and Geometrical Dependence of Water
Oxidation Activity in Co-Based Layered Hydroxides. ACS Catal..

[13] Sanchis-Gual R., Jaramillo-Hernández C., Hunt D., Seijas-Da
Silva Á., Mizrahi M., Marini C., Oestreicher V., Abellán G. (2024). Influence of Crystallographic Structure and Metal Vacancies
on the Oxygen Evolution Reaction Performance of Ni-based Layered Hydroxides**. Chem. – Eur. J..

[14] Jaramillo-Hernández C., Seijas-Da Silva A., Abellán G. (2025). Crystallographic Phase-Dependent
Electrochemical Properties of Layered Hydroxides for Energy Applications. Eur. J. Inorg. Chem..

[15] Luan C., Liu G., Liu Y., Yu L., Wang Y., Xiao Y., Qiao H., Dai X., Zhang X. (2018). Structure Effects of
2D Materials on α-Nickel Hydroxide for Oxygen Evolution Reaction. ACS Nano.

[16] Seijas-Da
Silva A., Sanchis-Gual R., Carrasco J. A., Oestreicher V., Abellán G., Coronado E. (2020). Boosting the Supercapacitive Behavior
of CoAl Layered Double Hydroxides via Tuning the Metal Composition
and Interlayer Space. Batteries Supercaps.

[17] Carrasco J. A., Sanchis-Gual R., Silva A. S.-D., Abellán G., Coronado E. (2019). Influence of the Interlayer Space on the Water Oxidation
Performance in a Family of Surfactant-Intercalated NiFe-Layered Double
Hydroxides. Chem. Mater..

[18] Carrasco J. A., Romero J., Varela M., Hauke F., Abellán G., Hirsch A., Coronado E. (2016). Alkoxide-Intercalated
NiFe-Layered
Double Hydroxides Magnetic Nanosheets as Efficient Water Oxidation
Electrocatalysts. Inorg. Chem. Front..

[19] Carrasco J.
A., Harvey A., Hanlon D., Lloret V., McAteer D., Sanchis-Gual R., Hirsch A., Hauke F., Abellán G., Coleman J. N., Coronado E. (2019). Liquid Phase Exfoliation of Carbonate-Intercalated
Layered Double Hydroxides. Chem. Commun..

[20] Seijas-Da
Silva A., Oestreicher V., Coronado E., Abellán G. (2022). Influence
of Fe-Clustering on the Water Oxidation Performance of Two-Dimensional
Layered Double Hydroxides. Dalton Trans..

[21] He, J. ; Wei, M. ; Li, B. ; Kang, Y. ; Evans, D. G. ; Duan, X. Preparation of Layered Double Hydroxides. In Layered Double Hydroxides; Duan, X. ; Evans, D. G. , Eds.; Structure and Bonding; Springer-Verlag: Berlin/Heidelberg, 2006; Vol. 119, pp 89–119 10.1007/430_006.

[22] He S., An Z., Wei M., Evans D. G., Duan X. (2013). Layered Double Hydroxide-Based
Catalysts: Nanostructure Design and Catalytic Performance. Chem. Commun..

[23] Yu J., Wang Q., O’Hare D., Sun L. (2017). Preparation of Two
Dimensional Layered Double Hydroxide Nanosheets and Their Applications. Chem. Soc. Rev..

[24] Jaramillo-Hernández C., Oestreicher V., Mizrahi M., Abellán G. (2023). Upscaling
the Urea Method Synthesis of CoAl Layered Double Hydroxides. Beilstein J. Nanotechnol..

[25] Sanchis-Gual R., Seijas-Da Silva A., Coronado-Puchau M., Otero T. F., Abellán G., Coronado E. (2021). Improving the Onset Potential and Tafel Slope Determination
of Earth-Abundant Water Oxidation Electrocatalysts. Electrochim. Acta.

[26] Lee E., Park A.-H., Park H.-U., Kwon Y.-U. (2018). Facile Sonochemical
Synthesis of Amorphous NiFe-(Oxy)­Hydroxide Nanoparticles as Superior
Electrocatalysts for Oxygen Evolution Reaction. Ultrason. Sonochem..

[27] Liang X., Wang X., Zhuang J., Chen Y., Wang D., Li Y. (2006). Synthesis of Nearly
Monodisperse Iron Oxide and Oxyhydroxide Nanocrystals. Adv. Funct. Mater..

[28] Yang J., Liu H., Martens W. N., Frost R. L. (2010). Synthesis and Characterization of
Cobalt Hydroxide, Cobalt Oxyhydroxide, and Cobalt Oxide Nanodiscs. J. Phys. Chem. C.

[29] Lawrence M. J., Kolodziej A., Rodriguez P. (2018). Controllable Synthesis of Nanostructured
Metal Oxide and Oxyhydroxide Materials via Electrochemical Methods. Curr. Opin. Electrochem..

[30] Anantharaj S., Kundu S., Noda S. (2021). “The Fe Effect”:
A
Review Unveiling the Critical Roles of Fe in Enhancing OER Activity
of Ni and Co Based Catalysts. Nano Energy.

[31] Trotochaud L., Young S. L., Ranney J. K., Boettcher S. W. (2014). Nickel–Iron
Oxyhydroxide Oxygen-Evolution Electrocatalysts: The Role of Intentional
and Incidental Iron Incorporation. J. Am. Chem.
Soc..

[32] Garcia A. C., Touzalin T., Nieuwland C., Perini N., Koper M. T. M. (2019). Enhancement
of Oxygen Evolution Activity of Nickel Oxyhydroxide by Electrolyte
Alkali Cations. Angew. Chem., Int. Ed..

[33] Becker H., Murawski J., Shinde D. V., Stephens I. E. L., Hinds G., Smith G. (2023). Impact of Impurities on Water Electrolysis: A Review. Sustainable Energy Fuels.

[34] Li H., Zhang Y., Chen Y., Li Y., Li Z., Yang B., Zhang Q., Lu J., Lei L., Xu Z. J., Hou Y. (2025). Leveraging Iron in the Electrolyte
to Improve Oxygen Evolution Reaction Performance: Fundamentals, Strategies,
and Perspectives. Angew. Chem., Int. Ed..

[35] Zhang R., Pearce P. E., Duan Y., Dubouis N., Marchandier T., Grimaud A. (2019). Importance of Water Structure and Catalyst–Electrolyte
Interface on the Design of Water Splitting Catalysts. Chem. Mater..

[36] Cheraparambil H., Vega-Paredes M., Wang Y., Tüysüz H., Scheu C., Weidenthaler C. (2024). Deciphering the Role of Fe Impurities
in the Electrolyte Boosting the OER Activity of LaNiO _3_. J. Mater. Chem. A.

[37] Klaus S., Trotochaud L., Cheng M., Head-Gordon M., Bell A. T. (2016). Experimental and
Computational Evidence of Highly Active
Fe Impurity Sites on the Surface of Oxidized Au for the Electrocatalytic
Oxidation of Water in Basic Media. ChemElectroChem.

[38] Perini N., Ticianelli E. A. (2019). Oxygen Evolution on Gold: The Effects of Alkali-Metal
Cations and Iron Impurities from Alkaline Electrolytes. J. Catal..

[39] Salmanion M., Najafpour M. M. (2021). Oxygen-Evolution Reaction by Gold
and Cobalt in Iron
and Nickel Free Electrolyte. Int. J. Hydrogen
Energy.

[40] Burke M. S., Enman L. J., Batchellor A. S., Zou S., Boettcher S. W. (2015). Oxygen
Evolution Reaction Electrocatalysis on Transition Metal Oxides and
(Oxy)­Hydroxides: Activity Trends and Design Principles. Chem. Mater..

[41] El-Refaei S. M., Rauret D. L., Manjón A. G., Spanos I., Zeradjanin A., Dieckhöfer S., Arbiol J., Schuhmann W., Masa J. (2024). Ni-Xides (B, S, and
P) for Alkaline OER: Shedding Light on Reconstruction
Processes and Interplay with Incidental Fe Impurities as Synergistic
Activity Drivers. ACS Appl. Energy Mater..

[42] Kawashima K., Márquez-Montes R. A., Li H., Shin K., Cao C. L., Vo K. M., Son Y. J., Wygant B. R., Chunangad A., Youn D. H., Henkelman G., Ramos-Sánchez V. H., Mullins C. B. (2021). Electrochemical
Behavior of a Ni_3_ N OER Precatalyst in Fe-Purified Alkaline
Media: The Impact of Self-Oxidation and Fe Incorporation. Mater. Adv..

[43] Du Y., He X., Yan C., Hu Q., Zhang J., Yang F. (2025). Promoted Surface
Reconstruction of Amorphous Nickel Boride Electrocatalysts by Boron
Dissolution for Boosting the Oxygen Evolution Reaction. J. Mater. Chem. A.

[44] Márquez R. A., Kawashima K., Son Y. J., Castelino G., Miller N., Smith L. A., Chukwuneke C. E., Mullins C. B. (2023). Getting the Basics Right: Preparing
Alkaline Electrolytes
for Electrochemical Applications. ACS Energy
Lett..

[45] Chung D. Y., Lopes P. P., Martins P. F. B. D., He H., Kawaguchi T., Zapol P., You H., Tripkovic D., Strmcnik D., Zhu Y., Seifert S., Lee S., Stamenkovic V. R., Markovic N. M. (2020). Dynamic Stability of Active Sites
in Hydr­(Oxy)­Oxides for the Oxygen Evolution Reaction. Nat. Energy.

[46] Son Y. J., Kawashima K., Wygant B. R., Lam C. H., Burrow J. N., Celio H., Dolocan A., Ekerdt J. G., Mullins C. B. (2021). Anodized
Nickel Foam for Oxygen Evolution Reaction in Fe-Free and Unpurified
Alkaline Electrolytes at High Current Densities. ACS Nano.

[47] Oestreicher V., Jobbágy M. (2013). One Pot Synthesis of Mg2Al­(OH)­6Cl·1.5H2O Layered
Double Hydroxides: The Epoxide Route. Langmuir.

[48] Oestreicher V., Fábregas I., Jobbágy M. (2014). One-Pot Epoxide-Driven Synthesis
of M2Al­(OH)­6Cl·1.5H2O Layered Double Hydroxides: Precipitation
Mechanism and Relative Stabilities. J. Phys.
Chem. C.

[49] Arencibia N., Oestreicher V., A Viva F., Jobbágy M. (2017). Nanotextured
Alpha Ni­(Ii)–Co­(Ii) Hydroxides as Supercapacitive Active Phases. RSC Adv..

[50] Hunt D., Oestreicher V., Mizrahi M., Requejo F. G., Jobbágy M. (2020). Unveiling
the Occurrence of Co­(III) in NiCo Layered Electroactive Hydroxides:
The Role of Distorted Environments. Chem. –
Eur. J..

[51] Liang J., Ma R., Iyi N., Ebina Y., Takada K., Sasaki T. (2010). Topochemical
Synthesis, Anion Exchange, and Exfoliation of Co–Ni Layered
Double Hydroxides: A Route to Positively Charged Co–Ni Hydroxide
Nanosheets with Tunable Composition. Chem. Mater..

[52] Liu Z., Ma R., Osada M., Iyi N., Ebina Y., Takada K., Sasaki T. (2006). Synthesis, Anion Exchange,
and Delamination of Co–Al
Layered Double Hydroxide: Assembly of the Exfoliated Nanosheet/Polyanion
Composite Films and Magneto-Optical Studies. J. Am. Chem. Soc..

[53] Newville M. (2001). *IFEFFIT* :
Interactive XAFS Analysis and *FEFF* Fitting. J. Synchrotron Radiat..

[54] Ravel B., Newville M. (2005). *ATHENA*, *ARTEMIS*, *HEPHAESTUS* :
Data Analysis for X-Ray Absorption Spectroscopy
Using *IFEFFIT*. J. Synchrotron
Radiat..

[55] Rehr J. J., Kas J. J., Vila F. D., Prange M. P., Jorissen K. (2010). Parameter-Free
Calculations of X-Ray Spectra with FEFF9. Phys.
Chem. Chem. Phys..

[56] de
A A Soler-Illia G. J., Jobbágy M., Regazzoni A. E., Blesa M. A. (1999). Synthesis of Nickel Hydroxide by Homogeneous Alkalinization.
Precipitation Mechanism. Chem. Mater..

[57] Liang J., Ma R., Iyi N., Ebina Y., Takada K., Sasaki T. (2010). Topochemical
Synthesis, Anion Exchange, and Exfoliation of Co–Ni Layered
Double Hydroxides: A Route to Positively Charged Co–Ni Hydroxide
Nanosheets with Tunable Composition. Chem. Mater..

[58] Wang Q., O’Hare D. (2012). Recent Advances
in the Synthesis and Application of
Layered Double Hydroxide (LDH) Nanosheets. Chem.
Rev..

[59] Abellán, G. ; Carrasco, J. A. ; Coronado, E. Layered Double Hydroxide Nanocomposites Based on Carbon Nanoforms. In Layered Double Hydroxide Polymer Nanocomposites; Elsevier, 2020; pp 411–460 10.1016/B978-0-08-101903-0.00010-6.

[60] Fan G., Li F., Evans D. G., Duan X. (2014). Catalytic Applications of Layered
Double Hydroxides: Recent Advances and Perspectives. Chem. Soc. Rev..

[61] Sarfraz M., Shakir I. (2017). Recent Advances in
Layered Double Hydroxides as Electrode
Materials for High-Performance Electrochemical Energy Storage Devices. J. Energy Storage.

[62] Taibi M., Ammar S., Jouini N., Fiévet F., Molinié P., Drillon M. (2002). Layered Nickel Hydroxide Salts: Synthesis,
Characterization and Magnetic Behaviour in Relation to the Basal Spacing. J. Mater. Chem..

[63] Taibi M., Jouini N., Rabu P., Ammar S., Fiévet F. (2014). Lamellar Nickel
Hydroxy-Halides: Anionic Exchange Synthesis, Structural Characterization
and Magnetic Behavior. J. Mater. Chem. C.

[64] Liu Z., Ma R., Osada M., Takada K., Sasaki T. (2005). Selective and Controlled
Synthesis of α- and β-Cobalt Hydroxides in Highly Developed
Hexagonal Platelets. J. Am. Chem. Soc..

[65] Oestreicher V., Dolle C., Hunt D., Fickert M., Abellán G. (2022). Room Temperature
Synthesis of Two-Dimensional Multilayer Magnets Based on α-CoII
Layered Hydroxides. Nano Mater. Sci..

[66] Oestreicher V., Hunt D., Dolle C., Borovik P., Jobbágy M., Abellán G., Coronado E. (2021). The Missing Link in the Magnetism
of Hybrid Cobalt Layered Hydroxides: The Odd–Even Effect of
the Organic Spacer. Chem. – Eur. J..

[67] Jobbágy M., Soler-Illia G. J. D. A. A., Regazzoni A. E., Blesa M. A. (1998). Synthesis of Copper­(II)-Containing
Nickel­(II) Hydroxide
Particles as Precursors of Copper­(II)-Substituted Nickel­(II) Oxides. Chem. Mater..

[68] Wang H., Gao J., Li Z., Ge Y., Kan K., Shi K. (2012). One-Step Synthesis
of Hierarchical α-Ni­(OH)­2 Flowerlike Architectures and Their
Gas Sensing Properties for NOx at Room Temperature. CrystEngComm.

[69] Bishwanathan S., Kaushik N., Oberoi S. K., Gupta P. K. (2025). Dual Engineering
of Electronic Structure and Lattice Strain via Ce Doping in NiMn-LDH
for Oxygen Evolution Reaction. ChemistrySelect.

[70] Iqbal A., Sabouni H., Hamdan N. M. (2024). In-Situ Grown Ternary Metal hydroxides@3D
Oriented Crumpled V2C MXene Sheets for Improved Electrocatalytic Oxygen
Evolution Reaction. Heliyon.

[71] Abdpour S., Fetzer M. N. A., Oestreich R., Beglau T. H. Y., Boldog I., Janiak C. (2024). Bimetallic CPM-37­(Ni,Fe)
Metal–Organic Framework:
Enhanced Porosity, Stability and Tunable Composition. Dalton Trans..

[72] Ede S. R., Anantharaj S., Kumaran K. T., Mishra S., Kundu S. (2017). One Step Synthesis
of Ni/Ni­(OH)_2_ Nano Sheets (NSs) and Their Application in
Asymmetric Supercapacitors. RSC Adv..

[73] Li J., Zhao W., Huang F., Manivannan A., Wu N. (2011). Single-Crystalline Ni­(OH)­2 and NiO
Nanoplatelet Arrays as Supercapacitor
Electrodes. Nanoscale.

[74] Anjaneyulu C., Kumar S. N., Kumar V. V., Naresh G., Bhargava S. K., Chary K. V. R., Venugopal A. (2015). Influence
of La on Reduction Behaviour
and Ni Metal Surface Area of Ni–Al2O3 Catalysts for COx Free
H2 by Catalytic Decomposition of Methane. Int.
J. Hydrogen Energy.

[75] Butenko E., Bish D., Abrosimova G., Kapustin A. (2013). Comparison of Sorption
Properties of Natural and Synthetic Takovites, Ni6Al2­(OH)­16CO3. 4H2O. Epitoanyag - J. Silic. Based Compos. Mater..

[76] Shao M., Zhang R., Li Z., Wei M., Evans D. G., Duan X. (2015). Layered Double Hydroxides toward
Electrochemical Energy Storage and
Conversion: Design, Synthesis and Applications. Chem. Commun..

[77] Gao L., Cui X., Sewell C. D., Li J., Lin Z. (2021). Recent Advances in
Activating Surface Reconstruction for the High-Efficiency Oxygen Evolution
Reaction. Chem. Soc. Rev..

[78] Anantharaj S., Noda S. (2022). Dos and Don’ts
in Screening Water Splitting Electrocatalysts. Energy Adv..

[79] Wang Y., Arandiyan H., Dastafkan K., Li Y., Zhao C. (2020). Common Pitfalls
of Reporting Electrocatalysts for Water Splitting. Chem. Res. Chin. Univ..

[80] Song W., Xia C., Zaman S., Chen S., Xiao C. (2024). Advances in Stability
of NiFe-Based Anodes toward Oxygen Evolution Reaction for Alkaline
Water Electrolysis. Small.

[81] Bode H., Dehmelt K., Witte J. (1966). Zur Kenntnis
Der Nickelhydroxidelektrode—I.Über
Das Nickel (II)-Hydroxidhydrat. Electrochim.
Acta.

[82] Ferreira E. B., Jerkiewicz G. (2021). On the Electrochemical Reduction
of β-Ni­(OH)­2
to Metallic Nickel. Electrocatalysis.

[83] Straumanis M. E., Brakšs N. (1949). The Rate of Solution of High Purity Aluminum in Various
Bases. J. Electrochem. Soc..

[84] Chu D., Savinell R. F. (1991). Experimental Data
on Aluminum Dissolution in KOH Electrolytes. Electrochim. Acta.

[85] Dembowski M., Graham T. R., Reynolds J. G., Clark S. B., Rosso K. M., Pearce C. I. (2020). Influence of Soluble
Oligomeric Aluminum on Precipitation
in the Al–KOH–H _2_ O System. Phys. Chem. Chem. Phys..

[86] Peng L., Yang N., Yang Y., Wang Q., Xie X., Sun-Waterhouse D., Shang L., Zhang T., Waterhouse G. I. N. (2021). Atomic
Cation-Vacancy Engineering of NiFe-Layered Double Hydroxides for Improved
Activity and Stability towards the Oxygen Evolution Reaction. Angew. Chem., Int. Ed..

[87] Liu W., Yu J., Li T., Li S., Ding B., Guo X., Cao A., Sha Q., Zhou D., Kuang Y., Sun X. (2024). Self-Protecting
CoFeAl-Layered Double Hydroxides Enable Stable and Efficient Brine
Oxidation at 2 A Cm–2. Nat. Commun..

[88] Liu Q., Chen K., Wang M., Fan H., Yan Z., Du X., Chen Y. (2024). In-Situ Construction of Cation Vacancies in Amphoteric-Metallic
Element-Doped NiFe-LDH as Ultrastable and Efficient Alkaline Hydrogen
Evolution Electrocatalysts at 1000 mA Cm^–2^. J. Colloid Interface Sci..

[89] Guo H., Zhang L., Ou D., Liu Q., Wu Z., Yang W., Fang Z., Shi Q. (2024). Zn-Leaching
Induced
Rapid Self-Reconstruction of NiFe-Layered Double Hydroxides for Boosted
Oxygen Evolution Reaction. Small.

[90] Zhao J., Zhang J., Li Z., Bu X. (2020). Recent Progress on
NiFe-Based Electrocatalysts for the Oxygen Evolution Reaction. Small.

[91] Bodhankar P. M., Sarawade P. B., Singh G., Vinu A., Dhawale D. S. (2021). Recent
Advances in Highly Active Nanostructured NiFe LDH Catalyst for Electrochemical
Water Splitting. J. Mater. Chem. A.

[92] Mohammed-Ibrahim J. (2020). A Review on
NiFe-Based Electrocatalysts for Efficient Alkaline Oxygen Evolution
Reaction. J. Power Sources.

[93] Carrasco J. A., Seijas-Da Silva A., Oestreicher V., Romero J., Márkus B. G., Simon F., Vieira B. J. C., Waerenborgh J. C., Abellán G., Coronado E. (2020). Fundamental Insights into the Covalent
Silane Functionalization of NiFe Layered Double Hydroxides. Chem. – Eur. J..

[94] Zeng F., Mebrahtu C., Liao L., Beine A. K., Palkovits R. (2022). Stability
and Deactivation of OER Electrocatalysts: A Review. J. Energy Chem..

[95] Han Y., Wang J., Liu Y., Li T., Wang T., Li X., Ye X., Li G., Li J., Hu W., Deng Y. (2024). Stability Challenges and Opportunities of NiFe-based Electrocatalysts
for Oxygen Evolution Reaction in Alkaline Media. Carbon Neutralization.

[96] Li H., Lin Y., Duan J., Wen Q., Liu Y., Zhai T. (2024). Stability
of Electrocatalytic OER: From Principle to Application. Chem. Soc. Rev..

[97] Bao F., Kemppainen E., Dorbandt I., Xi F., Bors R., Maticiuc N., Wenisch R., Bagacki R., Schary C., Michalczik U., Bogdanoff P., Lauermann I., Van De Krol R., Schlatmann R., Calnan S. (2021). Host, Suppressor, and
Promoter—The Roles of Ni and Fe on Oxygen Evolution Reaction
Activity and Stability of NiFe Alloy Thin Films in Alkaline Media. ACS Catal..

[98] Seijas-Da
Silva Á., Oestreicher V., Huck-Iriart C., Mizrahi M., Hunt D., Ferrari V., Abellán G. (2024). Enhancing
the Supercapacitive Behaviour of Cobalt Layered Hydroxides by 3D Structuring
and Halide Substitution. Batteries Supercaps.

[99] Lv Q., Yao B., Zhang W., She L., Ren W., Hou L., Fautrelle Y., Lu X., Yu X., Li X. (2022). Controlled
Direct Electrodeposition of Crystalline NiFe/Amorphous NiFe-(Oxy)­Hydroxide
on NiMo Alloy as a Highly Efficient Bifunctional Electrocatalyst for
Overall Water Splitting. Chem. Eng. J..

[100] Hao Y., Li Y., Wu J., Meng L., Wang J., Jia C., Liu T., Yang X., Liu Z.-P., Gong M. (2021). Recognition
of Surface Oxygen Intermediates on NiFe Oxyhydroxide Oxygen-Evolving
Catalysts by Homogeneous Oxidation Reactivity. J. Am. Chem. Soc..

[101] Liu X., Jing S., Ban C., Wang K., Feng Y., Wang C., Ding J., Zhang B., Zhou K., Gan L., Zhou X. (2022). Dynamic Active
Sites in NiFe Oxyhydroxide upon Au Nanoparticles
Decoration for Highly Efficient Electrochemical Water Oxidation. Nano Energy.

[102] Pascuzzi M. E. C., Man A. J. W., Goryachev A., Hofmann J. P., Hensen E. J. M. (2020). Investigation of the Stability of
NiFe-(Oxy)­Hydroxide Anodes in Alkaline Water Electrolysis under Industrially
Relevant Conditions. Catal. Sci. Technol..

[103] Wei L., Du M., Zhao R., Lv F., Li L., Zhang L., Zhou D., Su J. (2022). High-Valence Mo Doping
for Highly Promoted Water Oxidation of NiFe (Oxy)­Hydroxide. J. Mater. Chem. A.

[104] Lai W., Ge L., Li H., Deng Y., Xu B., Ouyang B., Kan E. (2021). In Situ Raman
Spectroscopic Study
towards the Growth and Excellent HER Catalysis of Ni/Ni­(OH)­2 Heterostructure. Int. J. Hydrogen Energy.

[105] Hall D. S., Lockwood D. J., Poirier S., Bock C., MacDougall B. R. (2012). Raman and Infrared Spectroscopy of
α and β
Phases of Thin Nickel Hydroxide Films Electrochemically Formed on
Nickel. J. Phys. Chem. A.

[106] Mora M., Jiménez-Sanchidrián C., Rafael Ruiz J. (2014). Raman Spectroscopy Study of Layered-Double Hydroxides
Containing Magnesium and Trivalent Metals. Mater.
Lett..

[107] Louie M. W., Bell A. T. (2013). An Investigation of Thin-Film Ni–Fe
Oxide Catalysts for the Electrochemical Evolution of Oxygen. J. Am. Chem. Soc..

[108] Biesinger M. C., Payne B. P., Grosvenor A. P., Lau L. W. M., Gerson A. R., Smart R. St. C. (2011). Resolving Surface
Chemical States in XPS Analysis of First Row Transition Metals, Oxides
and Hydroxides: Cr, Mn, Fe, Co and Ni. Appl.
Surf. Sci..

[109] Demnitz M., Lamas Y. M., Barros R. L. G., de
Leeuw Den Bouter A., van der Schaaf J., de Groot M. T. (2024). Effect of Iron Addition
to the Electrolyte on Alkaline Water Electrolysis Performance. iScience.

[110] Marquez R. A., Kalokowski E., Espinosa M., Bender J. T., Son Y. J., Kawashima K., Chukwuneke C. E., Smith L. A., Celio H., Dolocan A., Zhan X., Miller N., Milliron D. J., Resasco J., Mullins C. B. (2024). Transition
Metal Incorporation: Electrochemical, Structure, and Chemical Composition
Effects on Nickel Oxyhydroxide Oxygen-Evolution Electrocatalysts. Energy Environ. Sci..

