# Supplementary material for: Fe Incorporation in Ni-Based Layered Hydroxides: Implications for Oxygen Evolution Electrocatalysis
Source: Inorg Chem. 2025 Nov 21;64(48):23360–76. doi: 10.1021/acs.inorgchem.5c02786 (PMC12690575; doi:10.1021/acs.inorgchem.5c02786)
Supplement: Supplementary file 1 [file ic5c02786_si_001.pdf]

Supporting Information

Fe Incorporation in Ni-Based Layered Hydroxides: Implications for Oxygen Evolution Electrocatalysis

*Camilo Jaramillo-Hernández[1], Alvaro Seijas-Da Silva[1], Vicente B. Vert[1], Martin Mizrahi[2,3], Antonio Leyva-Pérez[4] and Gonzalo Abellán[1]**

[1] Instituto de Ciencia Molecular (ICMol), Universidad de Valencia, Catedrático José Beltrán 2, 46980, Paterna, Valencia, Spain.

[2] Instituto de Investigaciones Fisicoquímicas Teóricas y Aplicadas (INIFTA) Departamento de Química, Facultad de Ciencias Exactas Universidad Nacional de La Plata, CCT La Plata- CONICET Diagonal 113 y 64, 1900, La Plata, Argentina.

[3] Facultad de Ingeniería, Universidad Nacional de La Plata calle 1 esq. 47,1900, La Plata, Argentina.

[4]Instituto de Tecnología Química. Universidad Politècnica de València–Agencia Estatal Consejo Superior de Investigaciones Científicas. Avda. de los Naranjos s/n, 46022, Valencia, Spain.

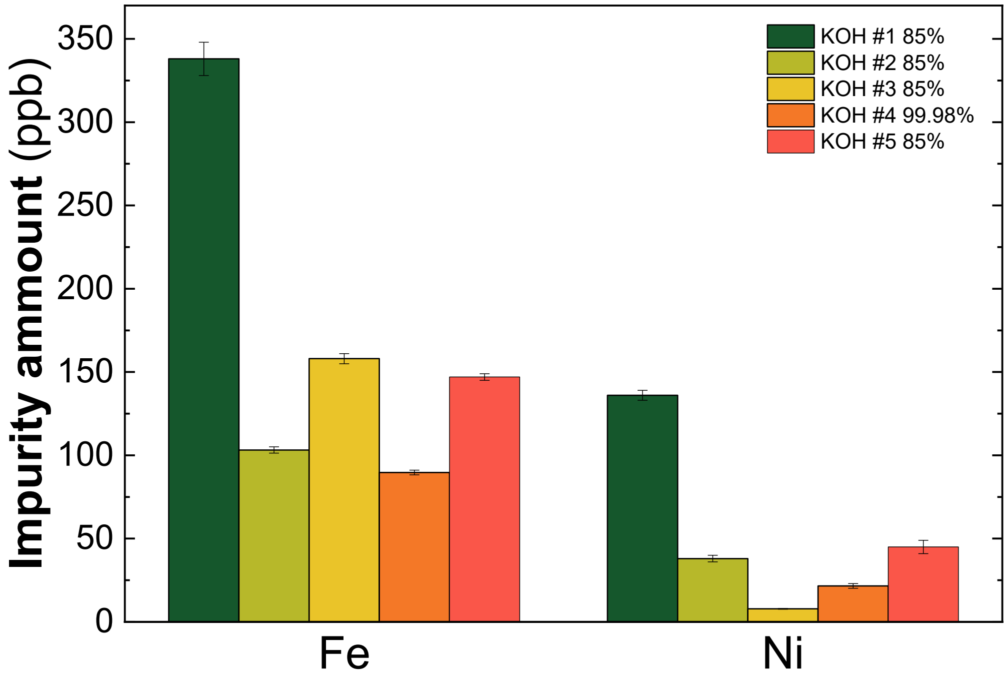

Figure S1. Fe and Ni comparison between different commercial KOH studied at 1M by ICP-MS..

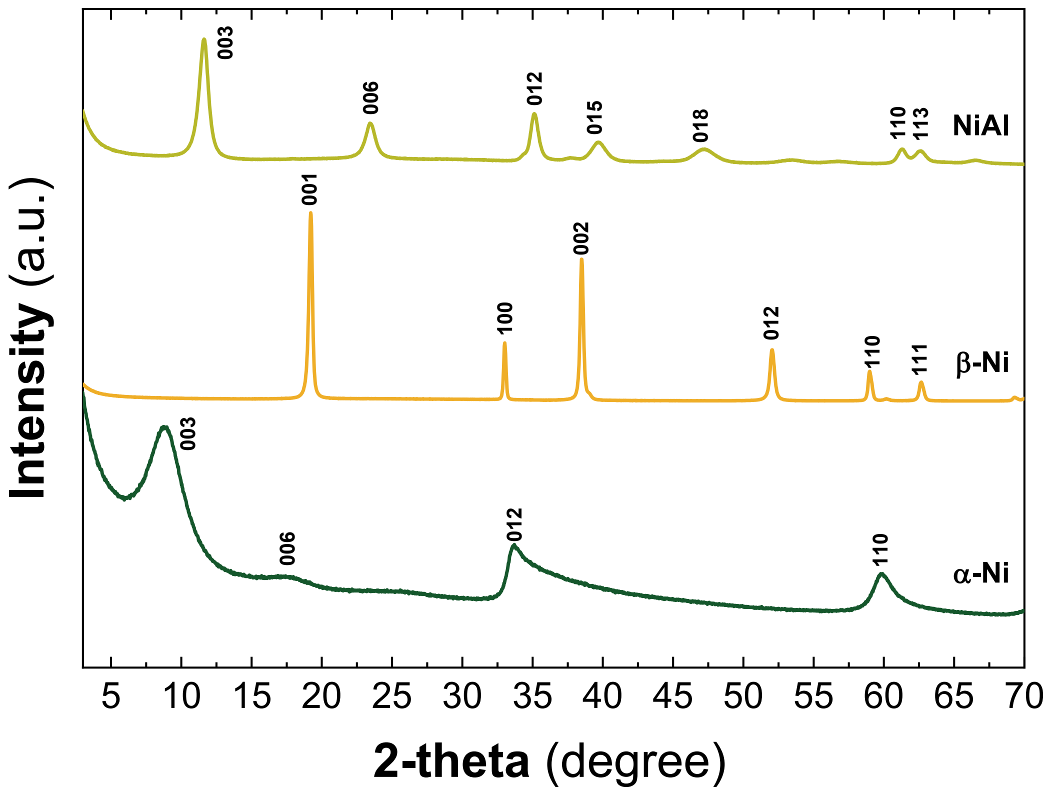

Figure S2. XRD diffractograms for the synthetized samples before any iron incorporation.

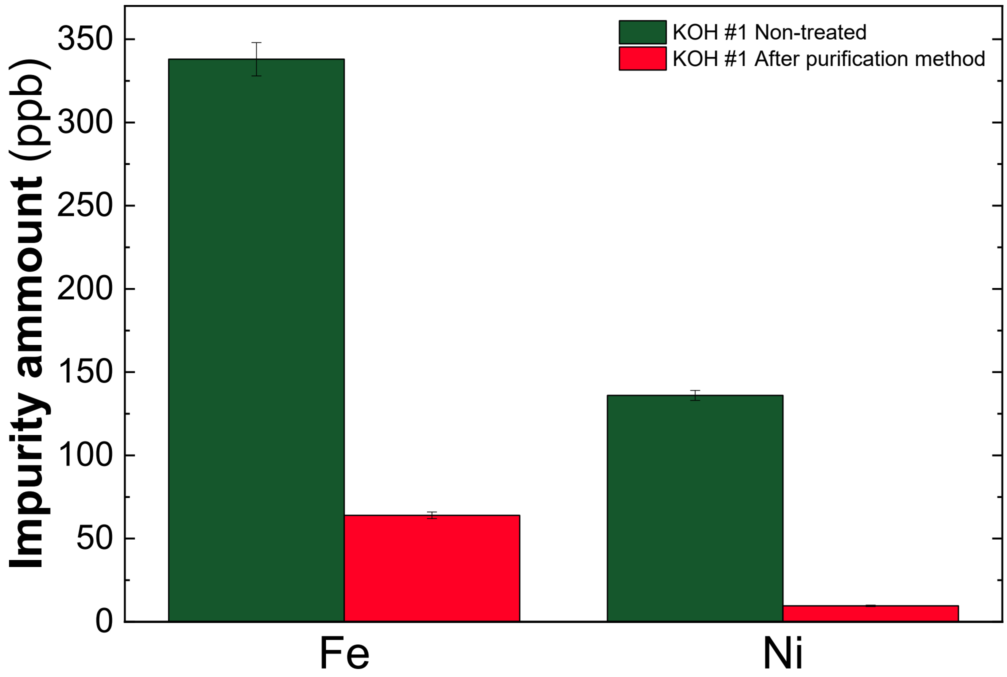

Figure S3. Fe and Ni amount comparison between KOH 1 before and after the electrochemical purification of the electrolyte.

SI Section 1

An electrochemical study was conducted on the aging treated samples as depicted in Figure S2. The trend observed in the electrochemical performance for the pure phases remains after these samples were aged under impure KOH, with the intensity values in the activation cycles are notably lower for both treated α-Ni LH and NiAl LDH. In the case of the NiAl LDH, the CVs exhibit considerable noise, which can be attributed to the intrinsically low conductivity of this material. This limitation may have been further affected by the aging treatment, resulting not only in very low current responses (<0.2 mA) but also in a weakened interaction between the material and the current collector, leading to noise at different points of the measurement. Nevertheless, the overall shape of the CV is consistent with the characteristic behaviour expected for NiAl LDHs.

This is also observed in the values obtained for all phases along the LSVs (Figure 2B) where the values of overpotential at 10 mA/cm2 are higher than those obtained for the non-treated materials (see inset in Figure 2B). Nonetheless, the same trend in performance remains (α-Ni LH> β-Ni LH> NiAl LDH). The overpotential values obtained for the different aged compounds at 10 mA/cm2 are as follows: 506 mV for the α-Ni LH, 597 mV for the β-Ni LH, and above 650 mV for the NiAl LDH.

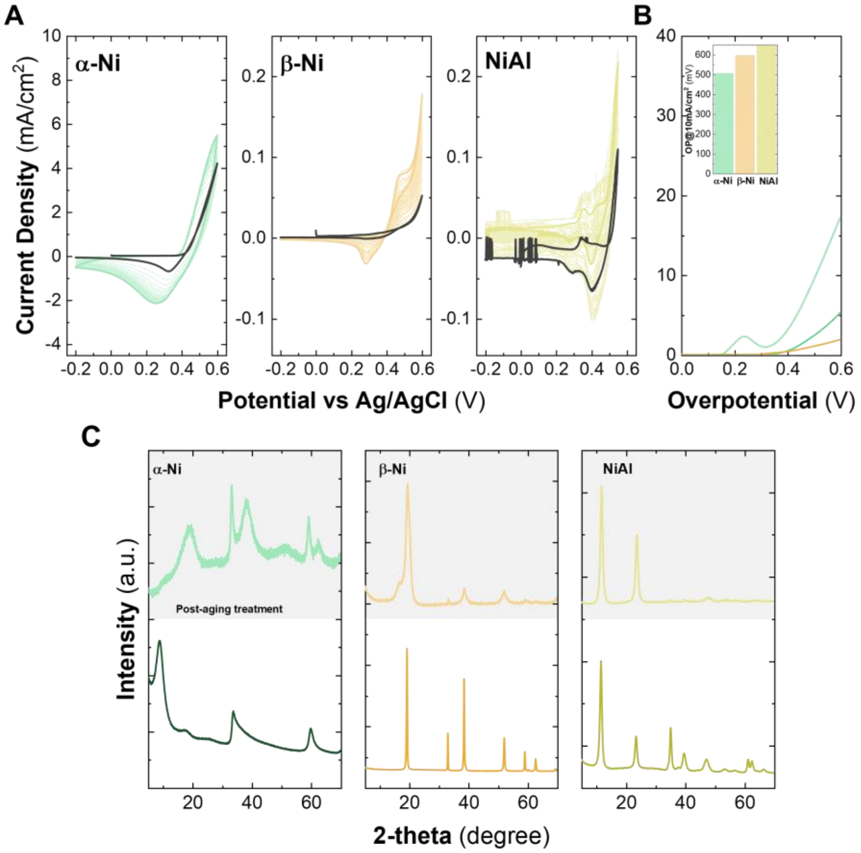

Figure S4. Electrochemical characterization in purified 1M KOH of the samples treated by aging incorporation: Cyclic Voltammetries for the samples α-Ni LH, β-Ni LH and NiAl LDH (A) Linear sweep voltammetry curves measured at 5 mV/s (B) and overpotential values required for a current density of 10 mA/cm^2 (inset in B) XRD diffractograms for the synthetized samples both before and after aging method of incorporation. (C).

Contrary to initial expectations, this incorporation does not lead to an improvement in electrochemical performance. The harsh mechanical treatment of the sample, involving shaking, centrifugation, and sonication in a basic medium, resulted in significant aggregation

and a subsequent decrease in electrochemical activity upon material recovery after filtration when compared to the as-synthesized materials.[57,58]

Powder X-ray Diffraction (PXRD) was performed on the samples after the aging treatment and before testing, to assess phase transformations. (See Figure S4). For α-Ni, there is a clear change in the crystallographic structure, with new peaks appearing around 20 º and 40 º, resembling those who are characteristic of a β-phase, indicating that the structure collapses. This transformation of the nickel hydroxide from α to β is a well-studied topotactic process, where the α-Ni, disposed in an alkali media during prolonged time can transform into a β-Ni.[59–63] For the β-Ni sample, a new peak close to 2-theta = 15º is observed, suggesting a small increase in the interlayer space. The peaks became broader and less defined, indicating that the aging treatment procedure affected the crystallinity, making the sample more amorphous and damaging the initial structure. Similarly, the NiAl sample did not show new peaks, but it exhibited a loss in crystallinity. The peaks remained at lower 2-theta values, indicating that, unlike the α-phase, the LDH structure did not collapse. In overall, these structural changes observed are completely related to the harsh conditions of the method (aging in basic media, strong agitations, etc). Moreover, these structural changes, for the α and β phases, where they are more pronounced, may explain why the electrochemical properties are affected resulting in worse values. In the case of NiAl, even though the change is not obvious, its initial poor performance seems to worsen even further.

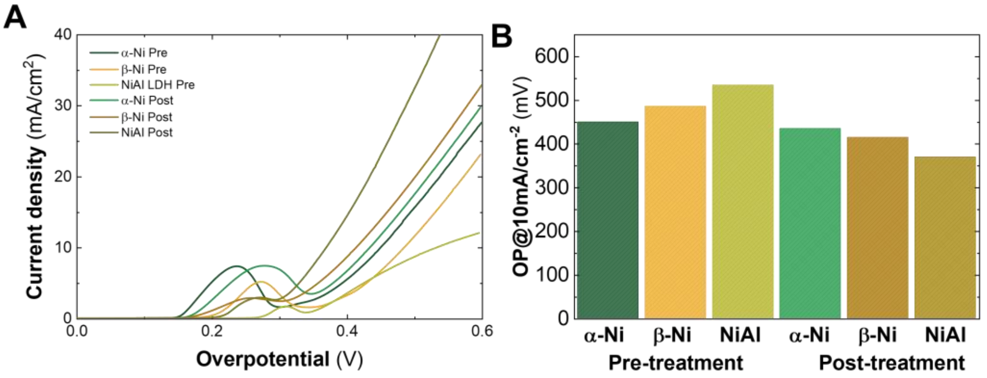

Figure S5. Linear sweep voltammetry curves measured in purified 1M KOH at 5 mV/s (A) and overpotential values required for a current density of 10 mA/cm^2 (B) comparing untreated samples with those subjected to electrochemical incorporation in non-purified 1M KOH.

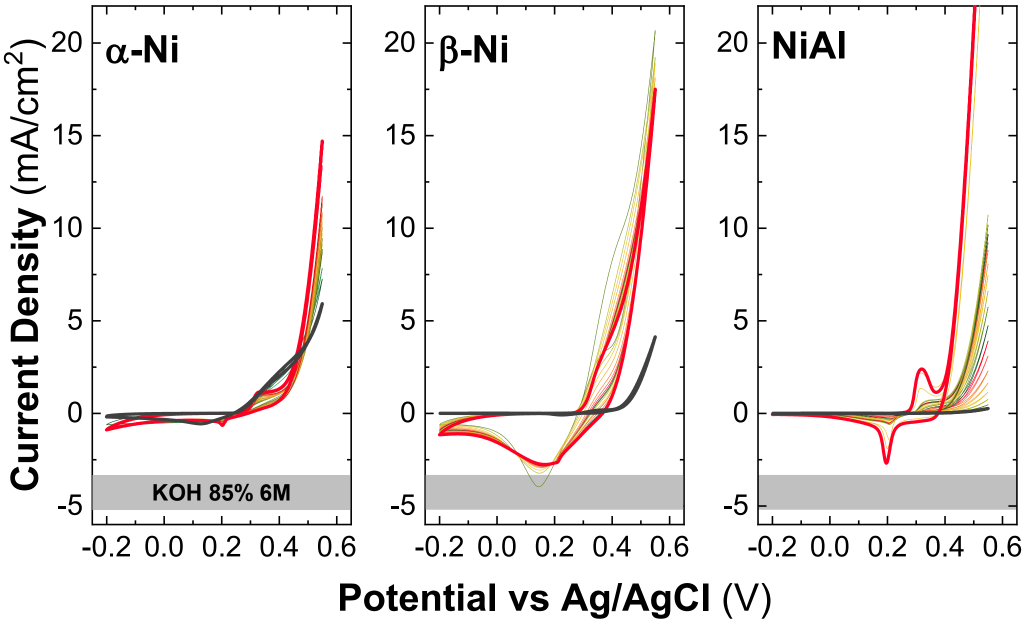

Figure S6. Voltamperograms for the electrochemical incorporation approach in a range of -0.2 to 0.55 V, in 6M KOH for samples α-Ni LH, β-Ni LH, and NiAl LDH. Greenish cycles correspond to the first ones while reddish ones belong to the last ones.

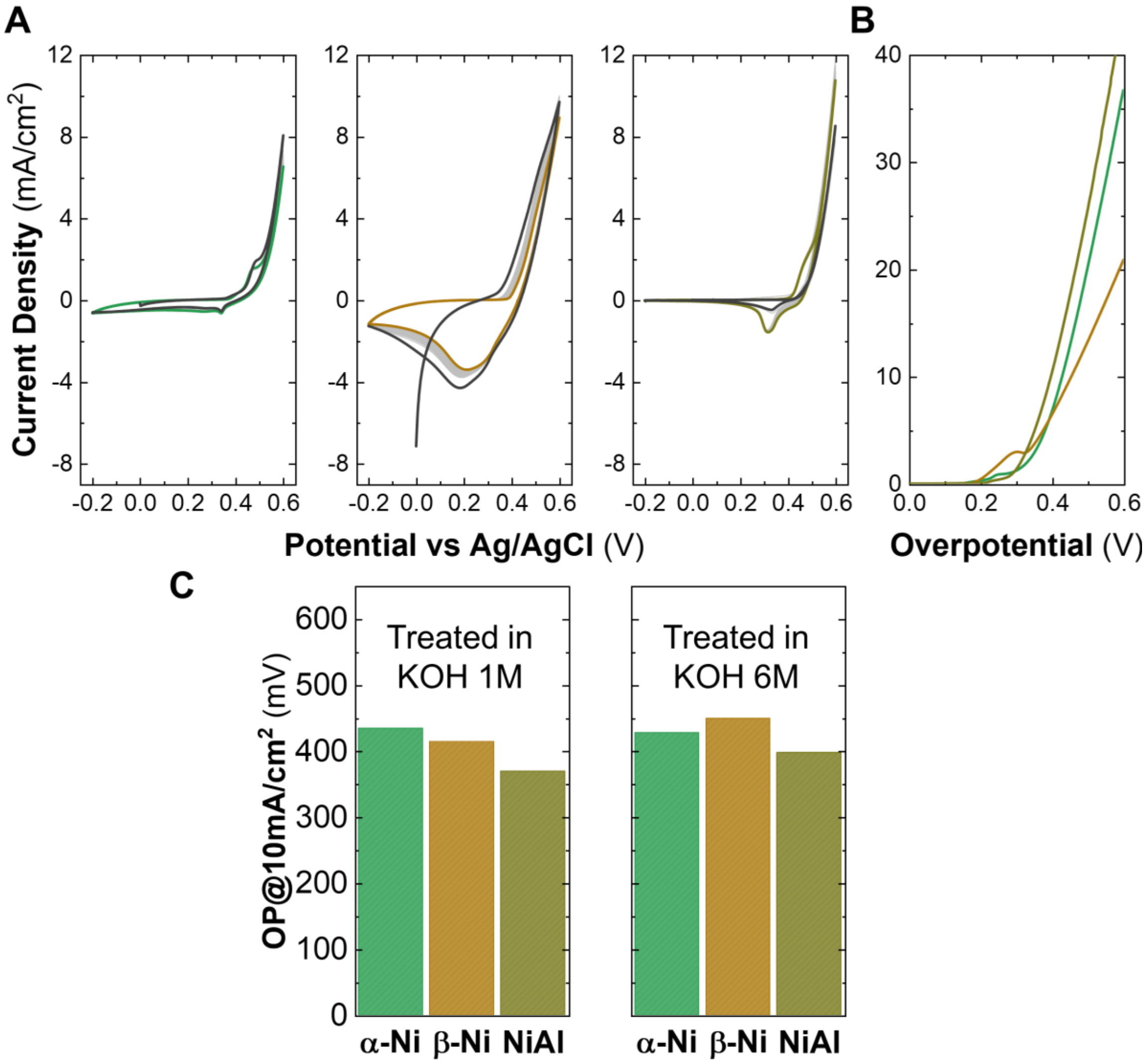

Figure S7. Electrochemical characterization of the samples treated by the electrochemical incorporation at 6M: Cyclic Voltammetries for the samples α-Ni LH, β-Ni LH and NiAl (A). Linear sweep voltammetry curves measured at 5 mV/s (B) Comparison on the overpotential values required for a current density of 10 mA/cm^2 for the electrochemical incorporation under 1M and 6M KOH. (C).

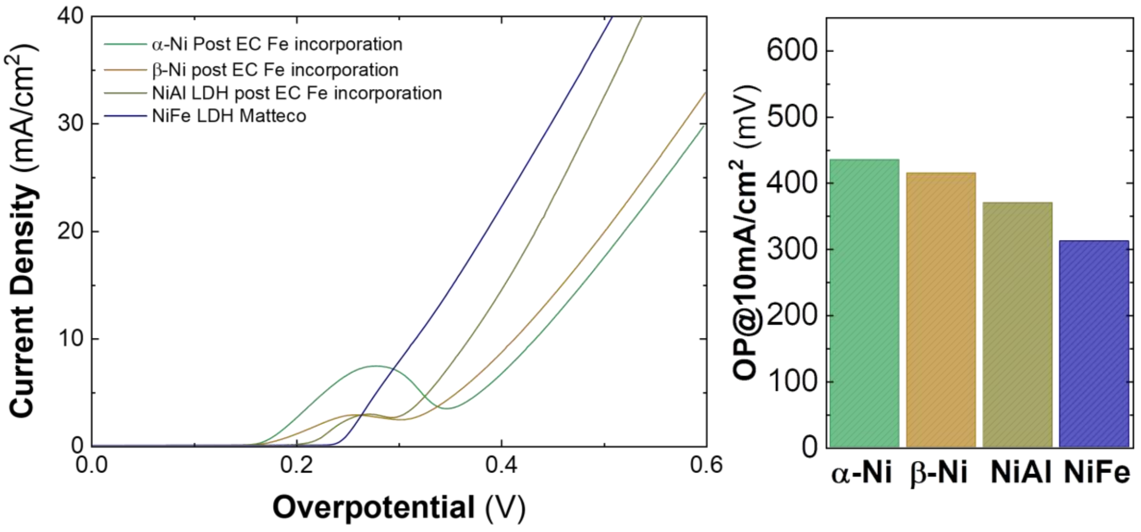

Figure S8. Comparison between the linear sweep voltammetry curves measured at 5 mV/s and the overpotential values required for a current density of 10 mA/cm^2 for the samples after the electrochemical incorporation and a commercial NiFe LDH. Samples measured were deposited on carbon paper substrates by spray coating.

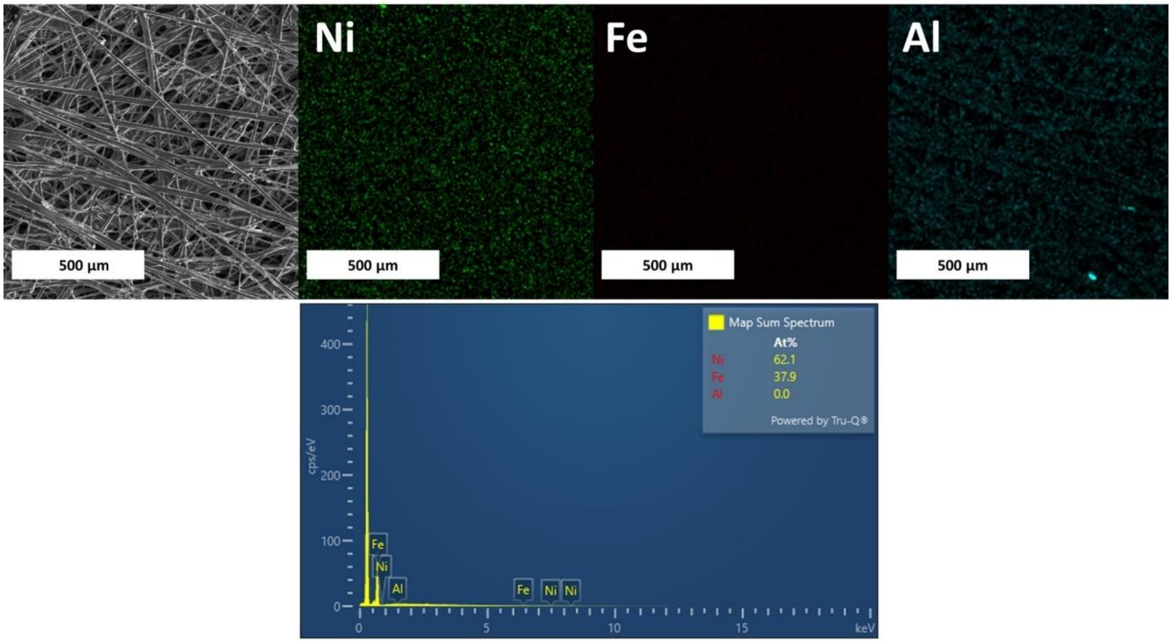

Figure S9. SEM-EDX Images and EDX spectrum for pristine carbon paper electrode.

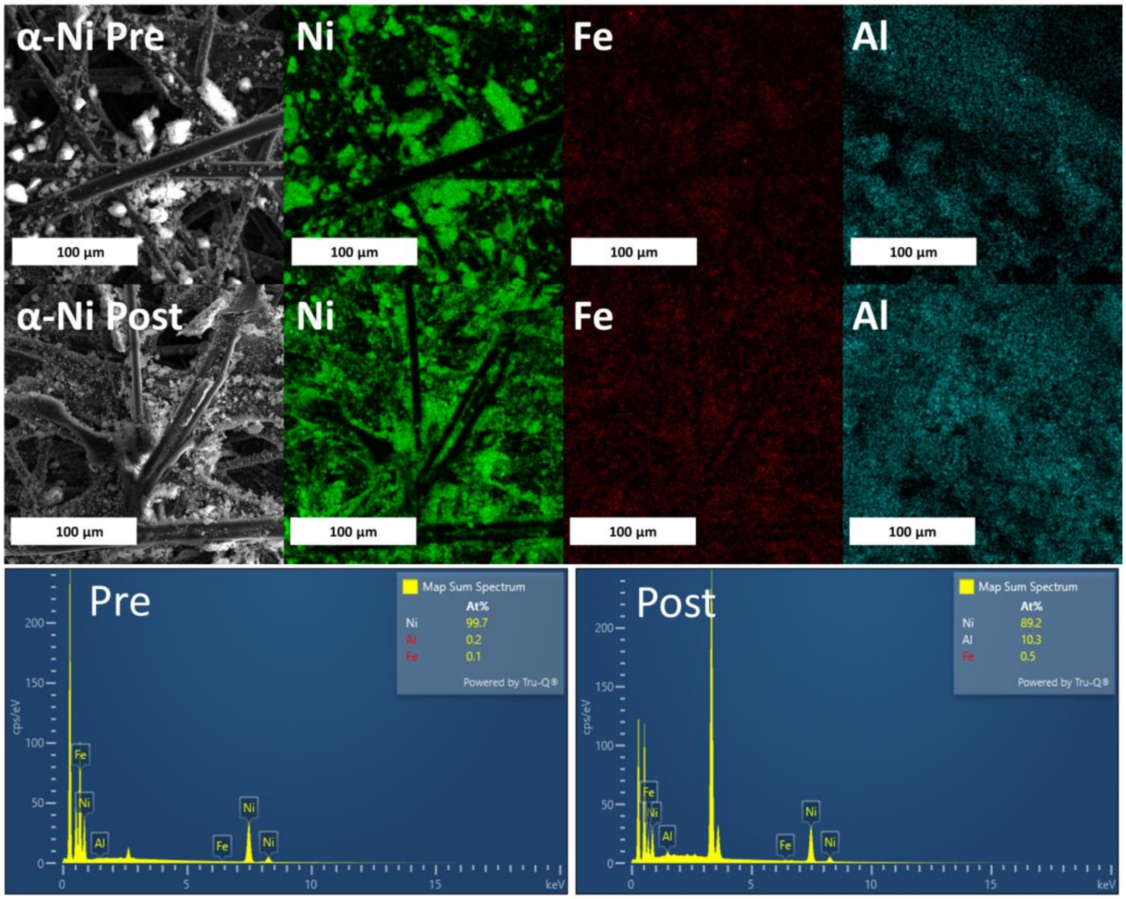

Figure S10. SEM-EDX Images and EDX spectrum for α-Ni-LH Sample: Pre- and Post-Electrochemical Incorporation, focusing on Ni and Fe.

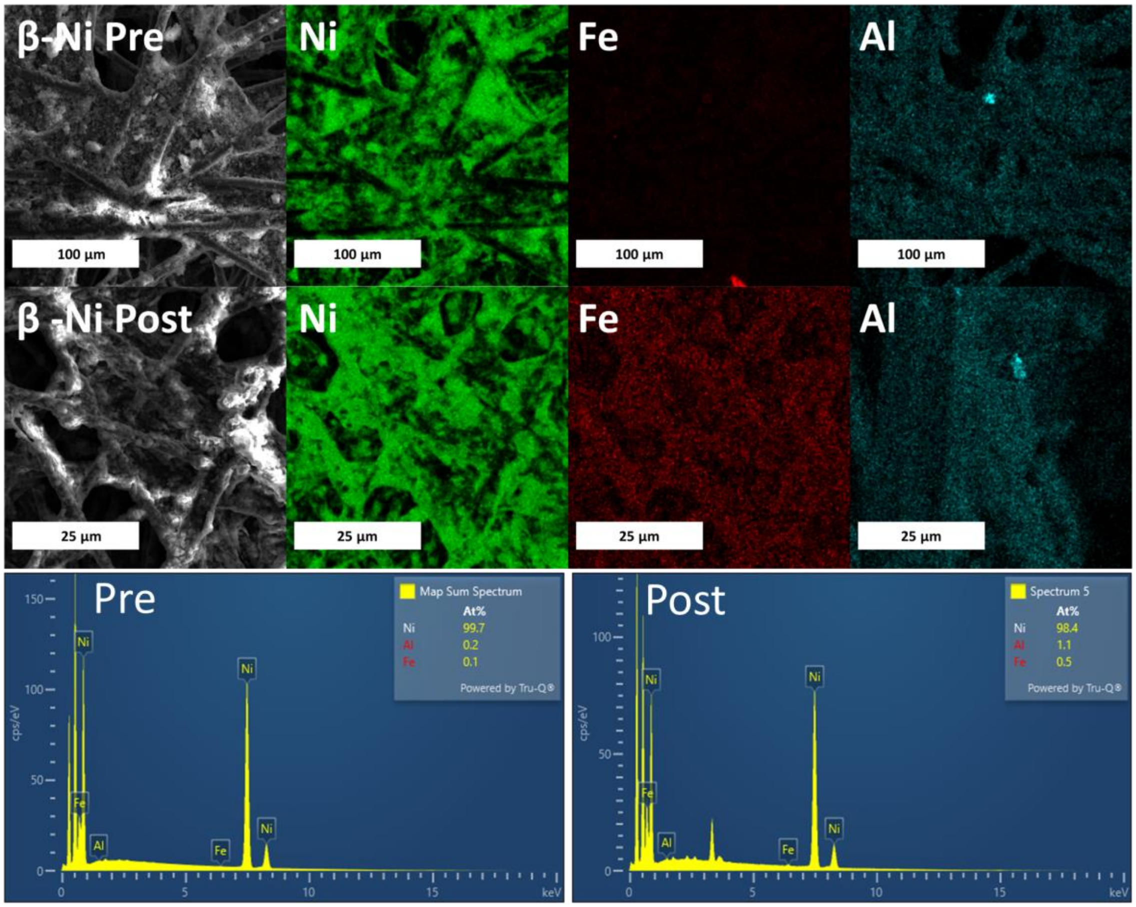

Figure S11 SEM-EDX Images and EDX spectrum for β-Ni-LH Sample: Pre- and Post-Electrochemical Incorporation, focusing on Ni and Fe.

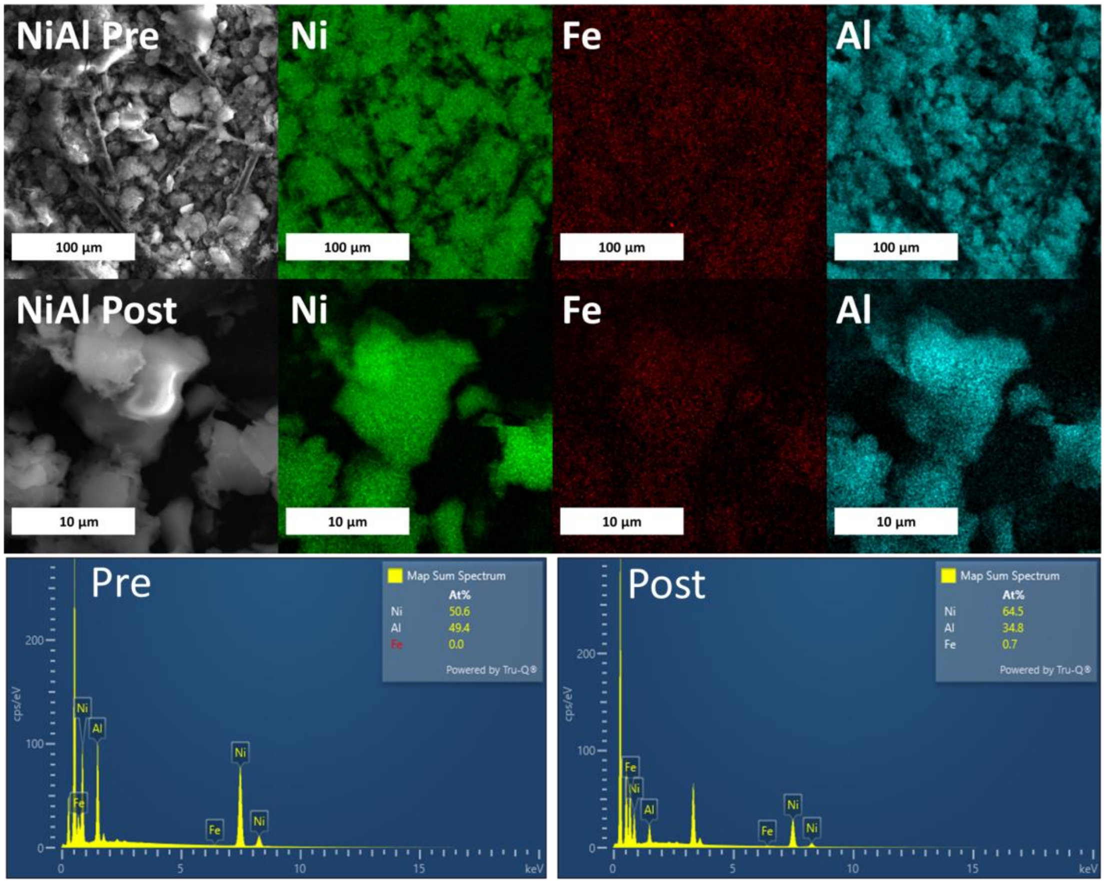

Figure S12. SEM-EDX Images and EDX spectrum for NiAl-LDH Sample: Pre- and Post-Electrochemical Incorporation, focusing on Ni and Fe.

Table S1. The percentage of Ni and Fe observed during SEM-EDX analysis for all the samples deposited on carbon paper, both before and after electrochemical incorporation.

Sample	Ni	Fe	Al
	%		
**α-Ni LH Pre**	99.7	0.1	0.2
**α-Ni LH Post**	89.2	0.5	10.3
**β-Ni LH Pre**	99.7	0.1	0.2
**β-Ni LH Post**	98.4	0.5	1.1
**NiAl LDH Pre**	50.6	0.0	49.4
**NiAl LDH Post**	64.5	0.7	34.8

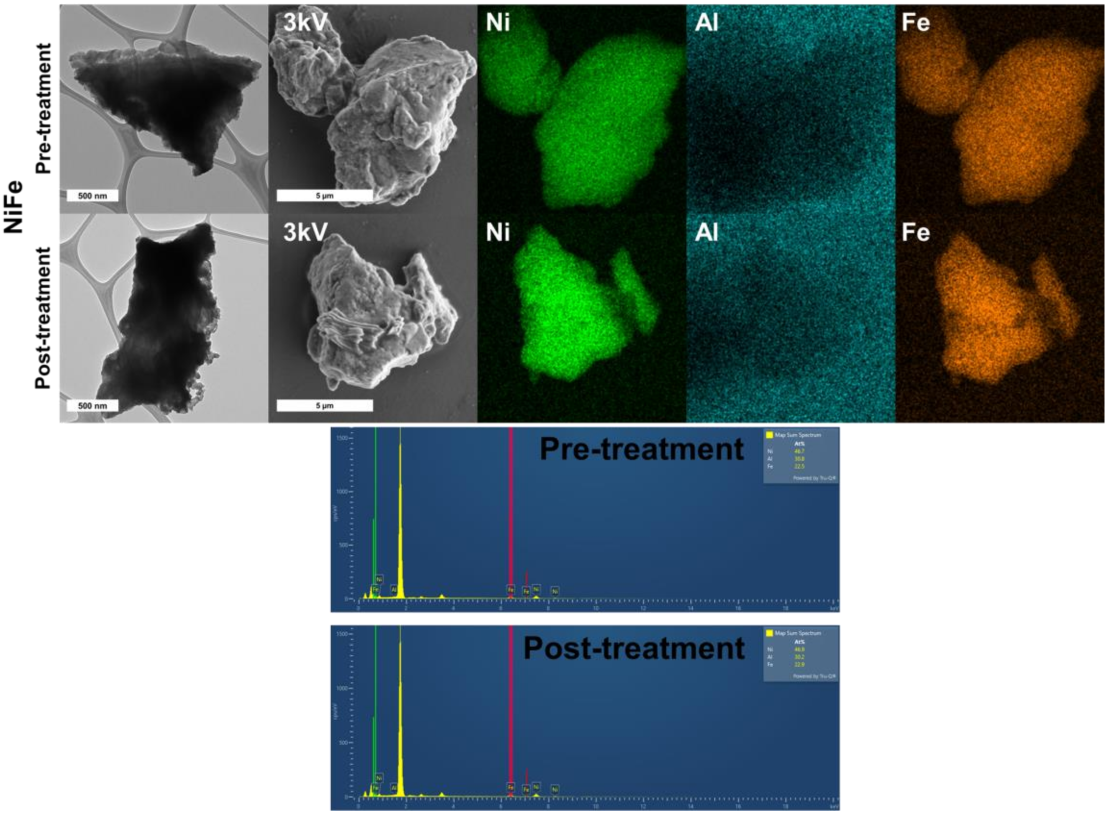

Figure S13. TEM & SEM images and EDX spectra of the NiFe LDHs recovered from the electrodes before and after the electrochemical treatment.

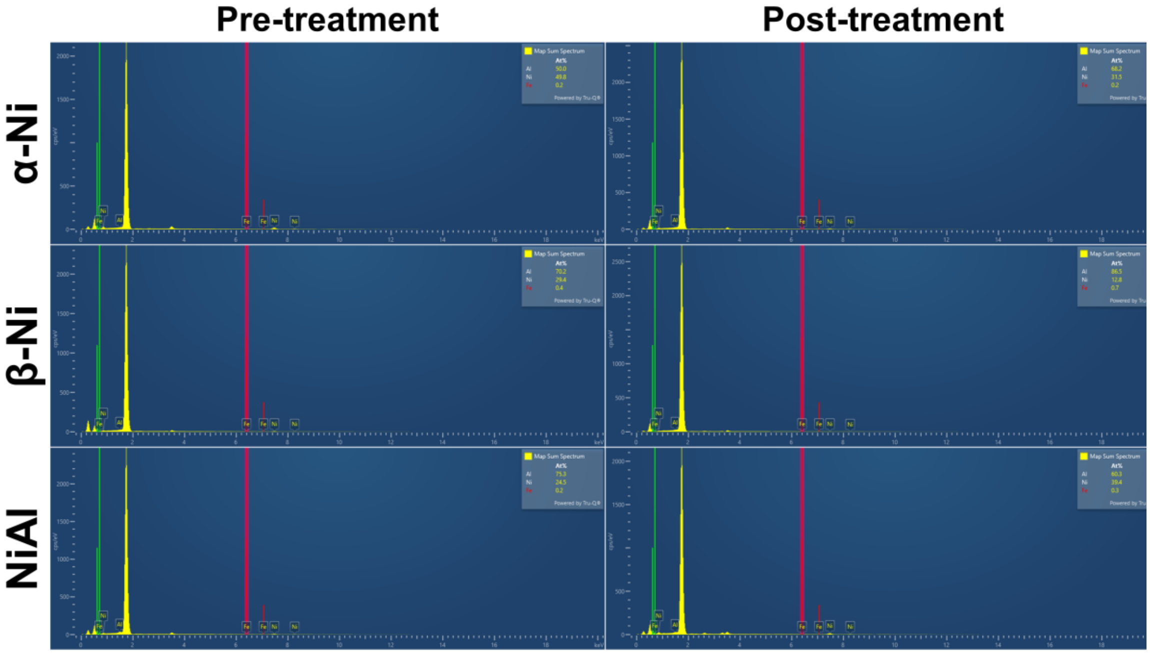

Figure S14. EDX spectra of the images exhibited in Figure 6.

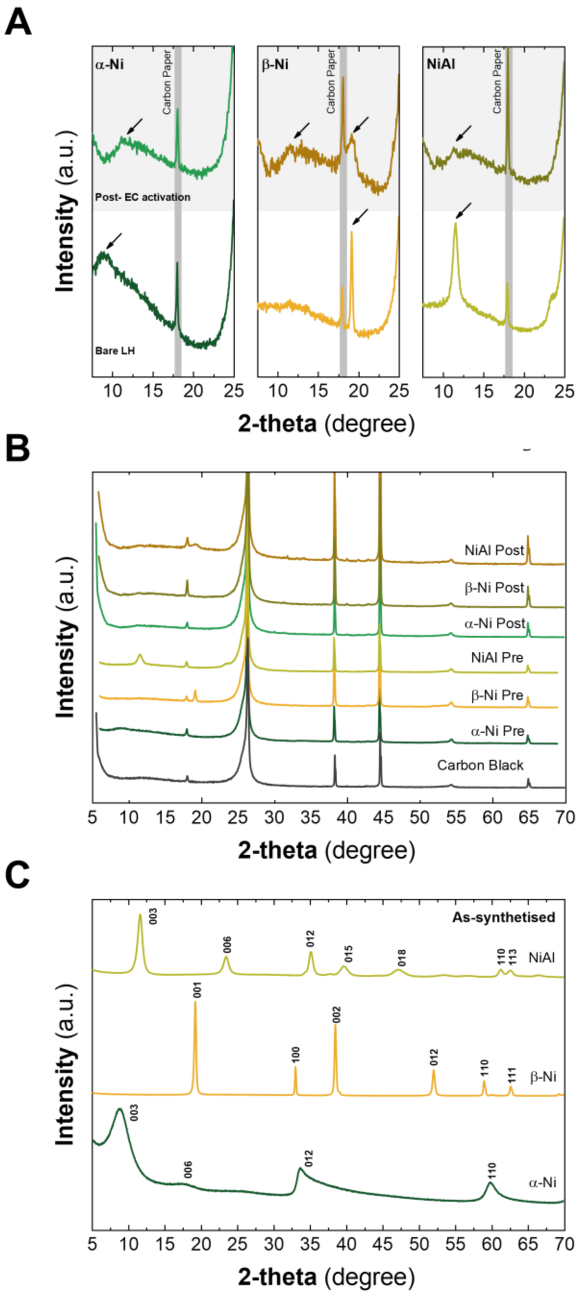

Figure S15. XRD diffractogram of the Ni-based layered hydroxide phases deposited in carbon paper, before and after the electrochemical incorporation approach (A), full diffractogram from 2-theta 5 to 70, presenting the diffractogram of the carbon paper (B) and XRD diffractograms for the synthetized samples (C)

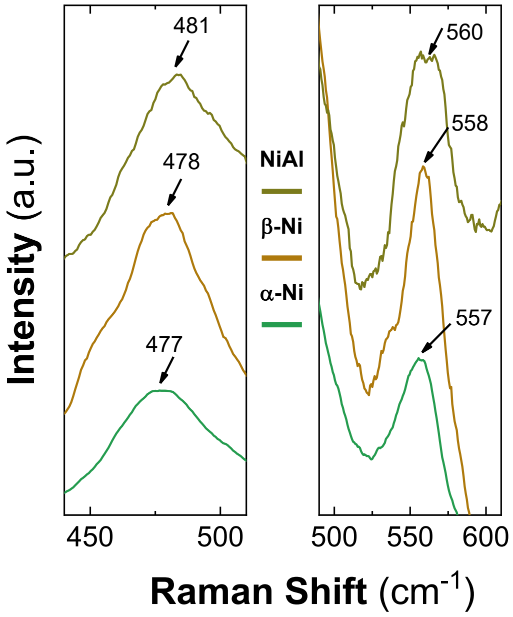

Figure S16. Raman spectra for the samples after the electrochemical Fe incorporation in the ranges of the E_g γ-NiOOH (left) and A_{1g} γ-NiOOH vibrations (right).

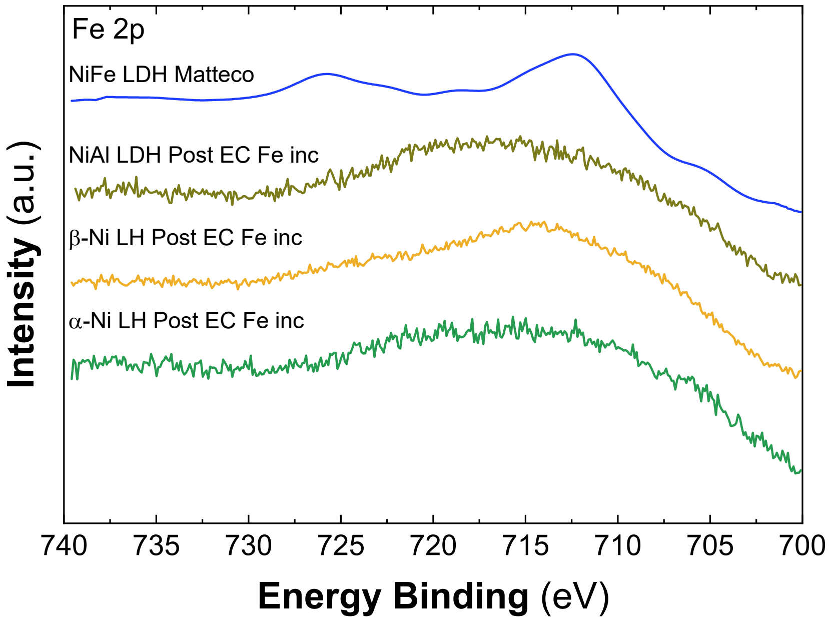

Figure S17. Comparison of the XPS signals on the Fe 2p region for the Ni-based layered hydroxide phases and a NiFe-LDH.

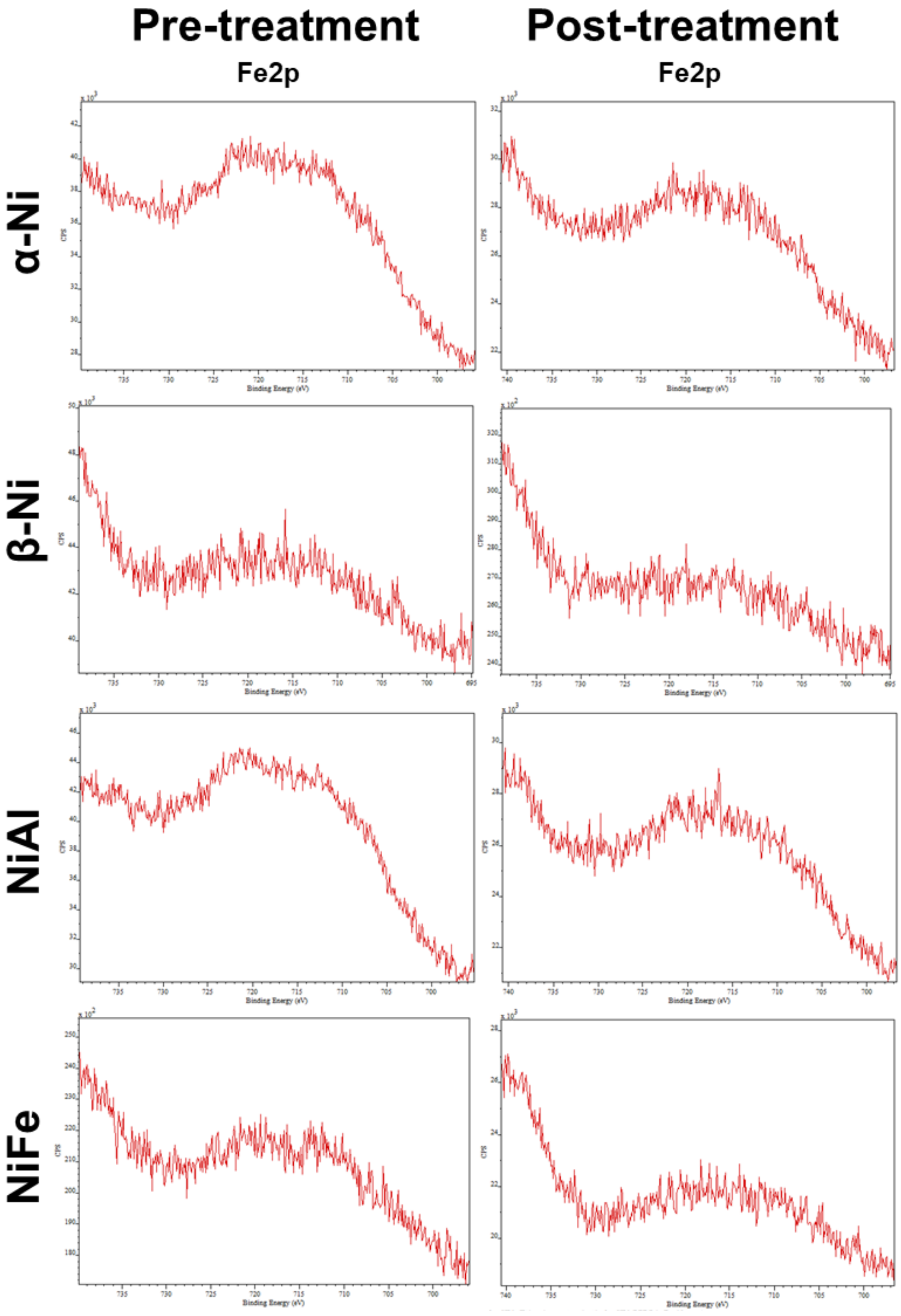

Figure S18. XPS measured on Kα Mg radiation on the Fe 2p region for the Ni-based layered hydroxide phases and a NiFe-LDH.

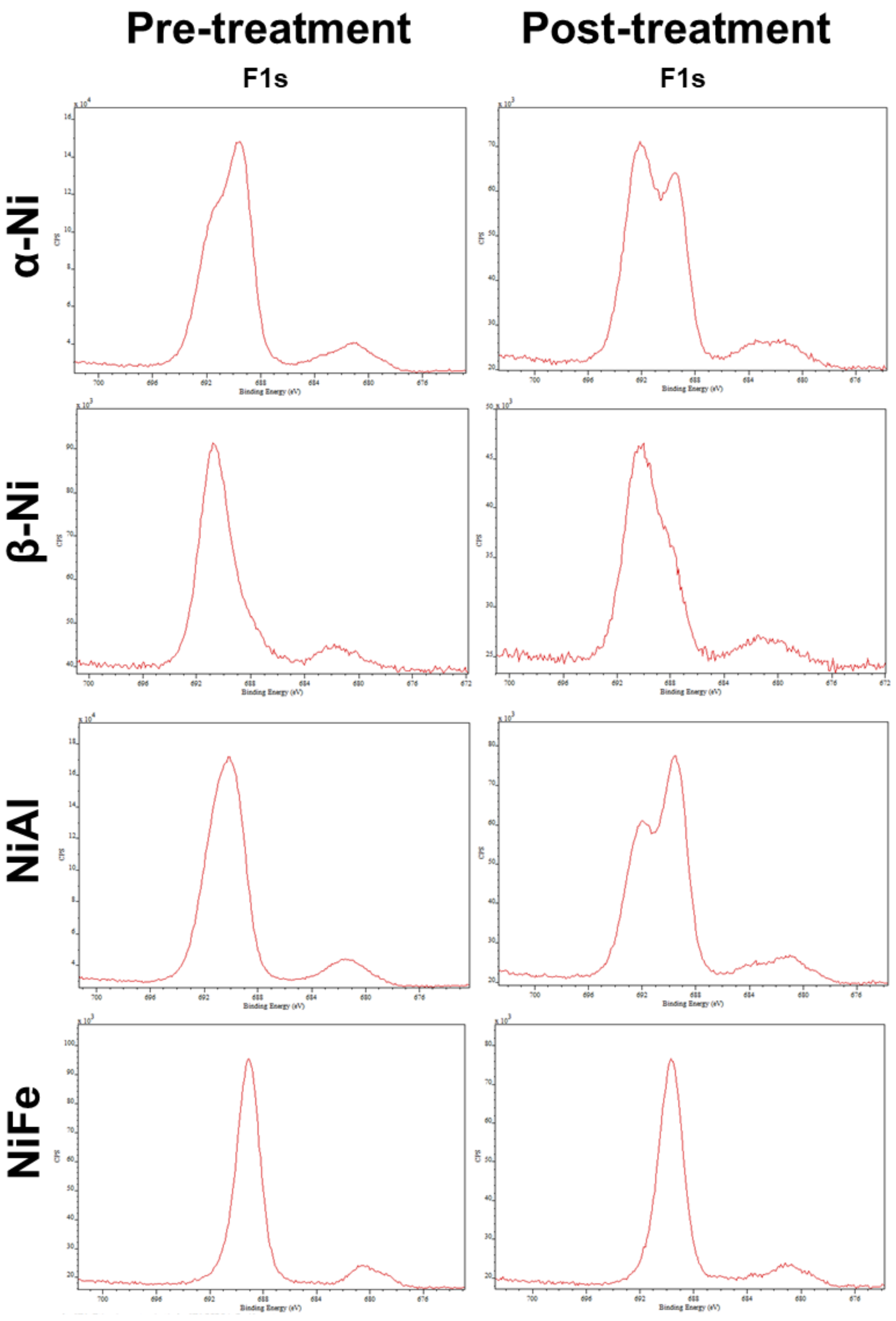

Figure S19. XPS measured on Kα Mg radiation on the F 1s region for the Ni-based layered hydroxide phases and a NiFe-LDH.

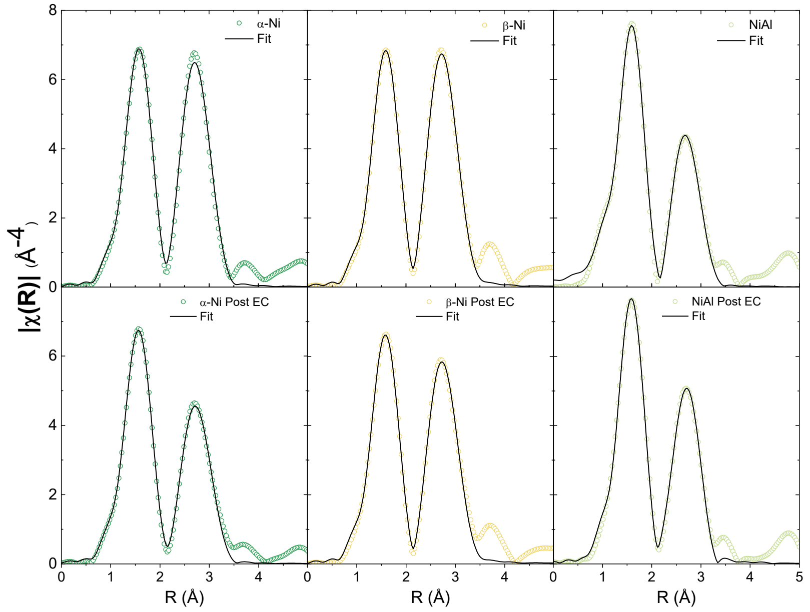

Figure S20. Fourier transform of the extracted EXAFS oscillations for the Ni LHs samples (symbol) and their corresponding fits (black line), measured at the Ni K-edge, before and after the electrochemical treatment

Table S2. Structural results from the EXAFS fitting (N: coordination number, R: interatomic distance and σ^2: Debye-Waller factor) at the Ni K-edge for the Ni-based LHs family.

**Sample**	***1st shell***			***2nd shell***					
	Ni-O			***Ni-Ni***			***Ni-Al***		
	N	***R*** (Å)	σ^2(Å^2)	***N***	***R*** (Å)	σ^2(Å^2)	***N***	***R*** (Å)	σ^2(Å^2)
α-Ni	5.8	2.07	0.006	5.2	3.09	0.007	-	-	-
β-Ni	5.8	2.09	0.006	5.4	3.11	0.007	-	-	-
NiAl	6.1	2.09	0.006	3.4	3.11	0.008	2.4	3.11	0.007
α-Ni post EC	5.8	2.07	0.006	4.2	3.10	0.008	-	-	-
β-Ni post EC	5.7	2.09	0.006	4.9	3.11	0.008	-	-	-
NiAl post EC	6.1	2.09	0.006	3.5	3.11	0.008	2.0	3.11	0.008

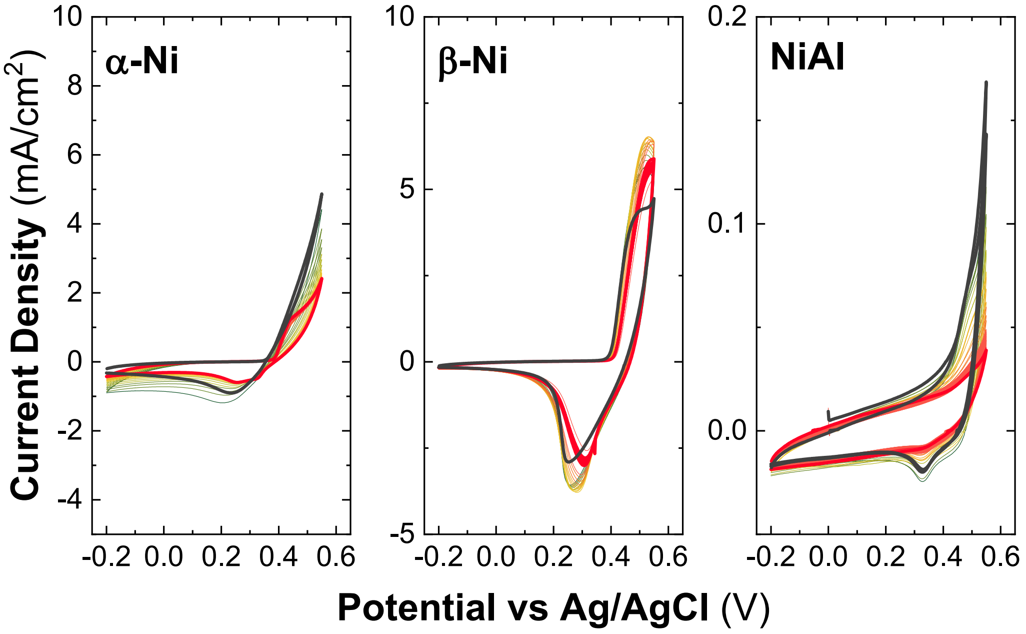

Figure S21. Voltamperograms for the electrochemical incorporation approach using KOH 2 in a range of -0.2 to 0.55 V, in 1M KOH for samples α-Ni LH, β-Ni LH, and NiAl LDH. Experiments were recorded using a different batch of KOH. Greenish cycles correspond to the first ones while reddish ones belong to the last ones

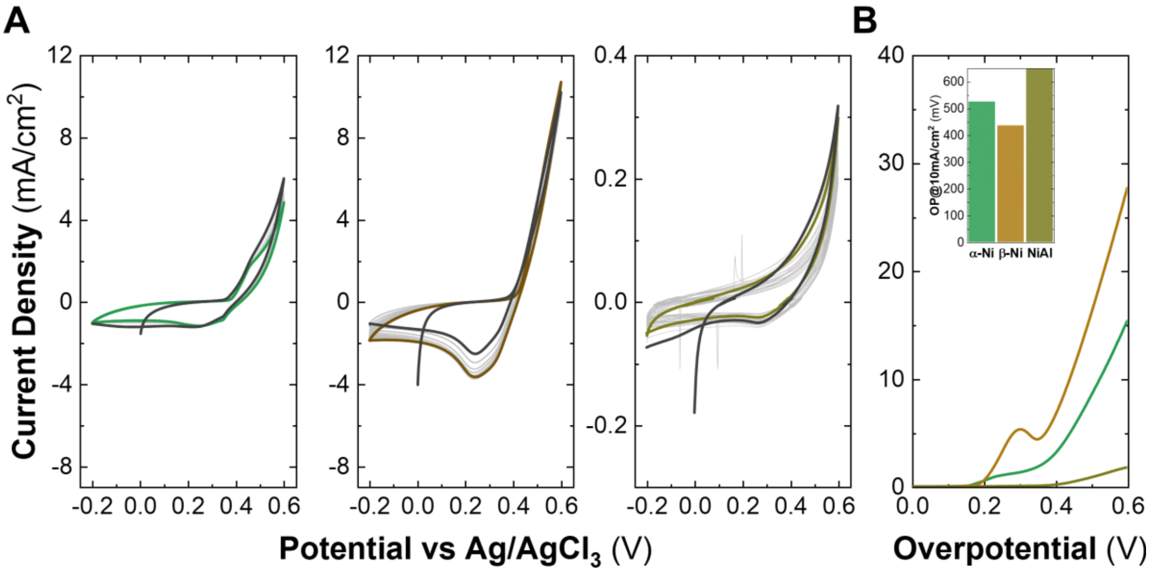

Figure S22. Electrochemical characterization of the samples treated by the electrochemical incorporation using KOH #2: Cyclic voltammetries for the samples α-Ni LH, β-Ni LH and NiAl (A). Linear sweep voltammetry curves measured at 5 mV/s (B) and overpotential values required for a current density of 10 mA/cm^2 (inset in B).

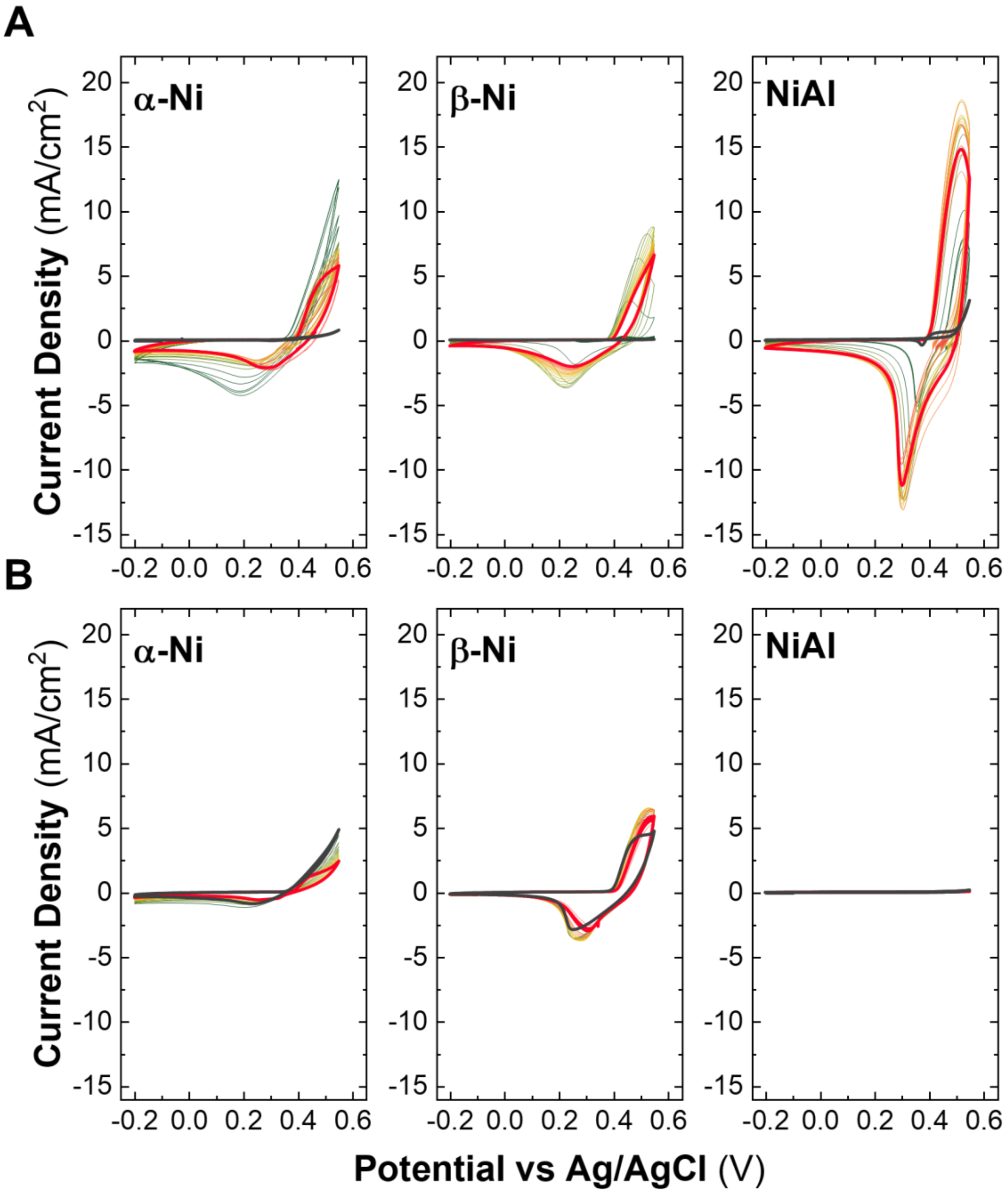

Figure S23. Comparison between the electrochemical incorporation. Cyclic voltammetries using KOH #1 (A) vs KOH #2 (B)

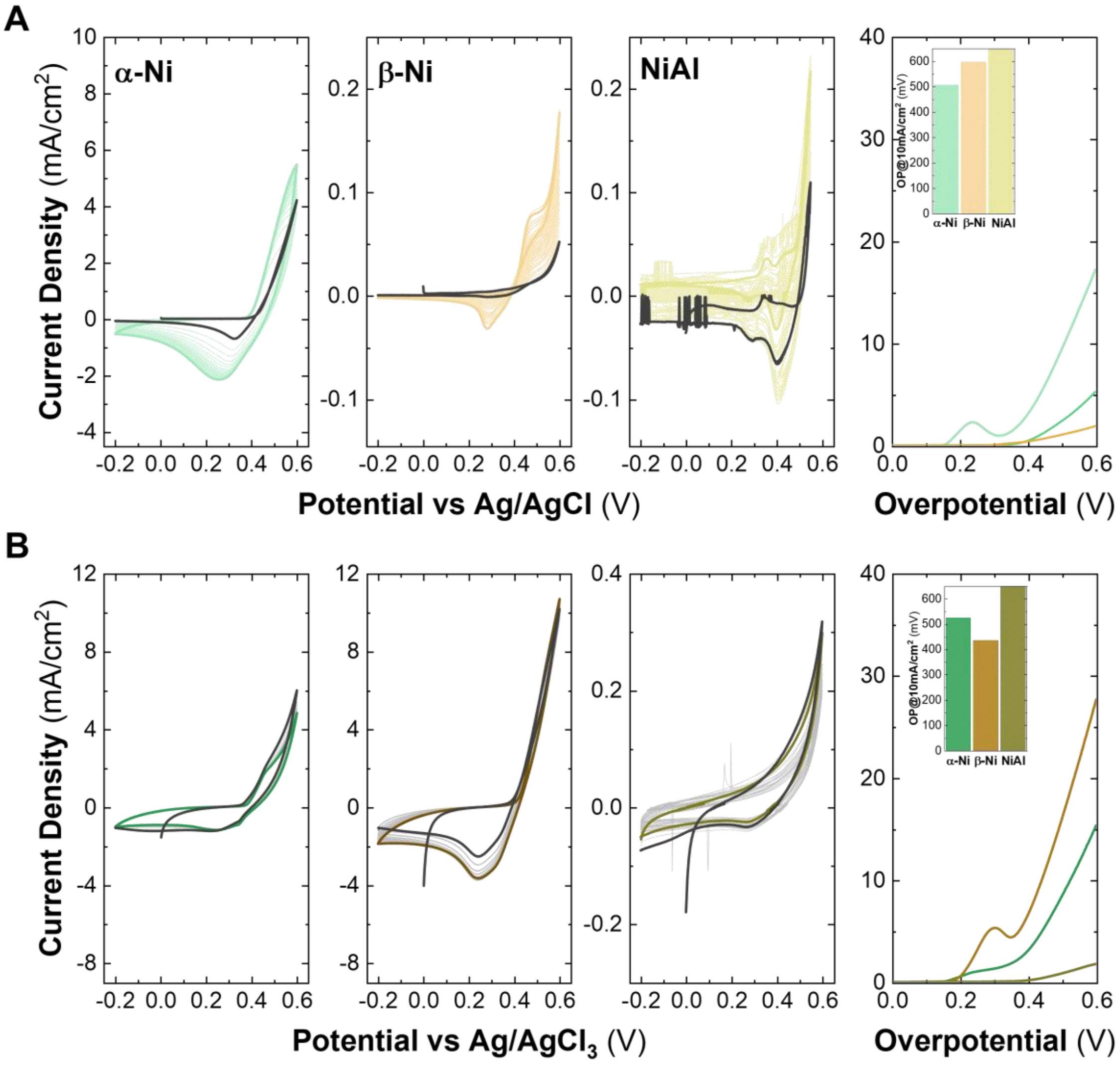

Figure S24. Comparison between the aging incorporation approach using KOH #1 (A) vs electrochemical incorporation approach using a KOH #2 (B)
